# Predictive network analysis identifies *JMJD6* and other potential key drivers in Alzheimer’s disease

**DOI:** 10.1038/s42003-023-04791-5

**Published:** 2023-05-15

**Authors:** Julie P. Merchant, Kuixi Zhu, Marc Y. R. Henrion, Syed S. A. Zaidi, Branden Lau, Sara Moein, Melissa L. Alamprese, Richard V. Pearse, David A. Bennett, Nilüfer Ertekin-Taner, Tracy L. Young-Pearse, Rui Chang

**Affiliations:** 1 Ann Romney Center for Neurologic Diseases, Brigham and Women’s Hospital and Harvard Medical School, Boston, MA USA; 2 The Center for Innovation in Brain Sciences, University of Arizona, Tucson, AZ USA; 3 Liverpool School of Tropical Medicine, Pembroke Place, Liverpool, Pembroke Place, L3 5QA UK; 4 Malawi–Liverpool—Wellcome Trust Clinical Research Programme, PO Box 30096, Blantyre, Malawi; 5 Arizona Research Labs, Genetics Core, University of Arizona, Tucson, AZ USA; 6 Rush Alzheimer’s Disease Center, Rush University Medical Center, Chicago, IL USA; 7 Department of Neuroscience, Mayo Clinic Florida, Jacksonville, FL USA; 8 Department of Neurology, Mayo Clinic Florida, Jacksonville, FL USA; 9 Harvard Stem Cell Institute, Harvard University, Boston, MA USA; 10 Department of Neurology, University of Arizona, Tucson, AZ USA; 11 INTelico Therapeutics LLC, Tucson, AZ USA; 12 PATH Biotech LLC, Tucson, AZ USA; 13 Present Address: Neuroscience Graduate Group, University of Pennsylvania Perelman School of Medicine, Philadelphia, PA USA

**Keywords:** Alzheimer's disease, Bayesian inference

## Abstract

Despite decades of genetic studies on late-onset Alzheimer’s disease, the underlying molecular mechanisms remain unclear. To better comprehend its complex etiology, we use an integrative approach to build robust predictive (causal) network models using two large human multi-omics datasets. We delineate bulk-tissue gene expression into single cell-type gene expression and integrate clinical and pathologic traits, single nucleotide variation, and deconvoluted gene expression for the construction of cell type-specific predictive network models. Here, we focus on neuron-specific network models and prioritize 19 predicted key drivers modulating Alzheimer’s pathology, which we then validate by knockdown in human induced pluripotent stem cell-derived neurons. We find that neuronal knockdown of 10 of the 19 targets significantly modulates levels of amyloid-beta and/or phosphorylated tau peptides, most notably *JMJD6*. We also confirm our network structure by RNA sequencing in the neurons following knockdown of each of the 10 targets, which additionally predicts that they are upstream regulators of REST and VGF. Our work thus identifies robust neuronal key drivers of the Alzheimer’s-associated network state which may represent therapeutic targets with relevance to both amyloid and tau pathology in Alzheimer’s disease.

## Abstract (web summary)

A network analysis on deconvoluted bulk transcriptomic data from human Alzheimer’s disease cohorts identifies several potential key disease drivers, including *JMJD6*.

## Introduction

Late-onset Alzheimer’s disease (LOAD) is the leading cause of dementia, which is characterized by progressive impairments in memory, cognition, and executive functions, along with behavioral and psychiatric symptoms including agitation, aggression, mood disorders, and psychosis^1^. The hallmark features of Alzheimer’s disease (AD) include pathological aggregation of extracellular plaques, composed of amyloid-β (Aβ) peptides, and intracellular neurofibrillary tangles, composed of hyperphosphorylated tau (p-tau) protein^2^, which lead to neuron death. Genome-wide association studies have implicated over 30 loci associated with AD risk^3–16^. In previous studies, we and others have shown that LOAD is a complex pathological process involving an interactive network of pathways among multiple cell types in the brain (neurons, microglia, astrocytes, etc.) influenced by genetic variation, aging, and environmental factors^17–20^. Implicated pathways include those involved in mitochondrial metabolism, response to unfolded proteins, immune response, phagocytosis, and synaptic transmission^21–24^. The complexity of these multi-modal networks highlights the necessity to study networks of molecular interactions by cell type and to identify cell type-specific pathways and key drivers in AD. In this study, we developed a multi-step pipeline using advanced computational systems biology approaches to construct robust data-driven neuron-specific network models of gene regulatory programs in brain regions affected by LOAD. For these analyses, we utilized whole-genome gene expression and whole-genome genotyping data from two independent cohorts in the Accelerating Medicines Partnership—Alzheimer’s Disease (AMP-AD) consortium: the Mayo RNAseq Study (herein MAYO) and the Religious Orders Study and Memory and Aging Project (herein ROSMAP).

We applied a deconvolution method to deconvolve bulk-tissue RNA sequencing (RNAseq) data from post-mortem brain regions and then derive the neuron-specific gene expression signal. Although single-cell RNA sequencing (scRNAseq) studies, including those from the recent Human Cell Atlas endeavor, have greatly advanced our understanding of cellular heterogeneity and the discovery of novel cell populations^25–35^ as well as spurred developments of various computational analysis tools^36^, network inference performance using scRNAseq data is still very poor. Due to the high volume of missing gene expression measures and the immaturity of current network methods dealing with these missing data, inferred network models using scRNAseq data yield a large amount of uncertainty^37,38^, thus limiting the application of scRNAseq data in network inference. Alternatively, deconvolution of bulk-tissue RNAseq data has become increasingly popular in recent years as a complementary solution to the missing values in scRNAseq data^39–51^, based on the core assumption that gene expression in bulk-tissue data is equal to the averaged gene expression of each cell type weighted by its relative population in the tissue. Deconvolution methods decompose bulk-tissue RNAseq data into gene expression of individual cell types by using cell type-specific biomarker genes to implicitly estimate relative cell populations in the tissue. After deconvolution, the variances of the deconvoluted gene expression of each cell type become orthogonal to each other and can be analyzed independently^52^.

To derive neuron-specific gene expression signals from the bulk-tissue RNAseq data from the MAYO and ROSMAP cohorts, we employed the population-specific expression analysis (PSEA) method of deconvolution^52^. Whereas other popular deconvolution methods such as Cibersort^43^, dtangle^40^, DSA^39^, or NNLS^53^ can only estimate cell fraction in a bulk-tissue sample, the PSEA method directly estimates cell type-specific residuals from bulk-tissue RNAseq data. Here, we demonstrated the robustness of the PSEA deconvolution method using random selection of neuronal biomarkers derived from scRNAseq studies^54–58^.

After deconvolution, we applied a cutting-edge systems biology approach^23,59,60^ to build causal network models of the neuronal component of AD by integrating the deconvoluted neuron-specific RNAseq data with the whole-genome genotype data from the MAYO and ROSMAP datasets. Bayesian networks^61^ are a long-standing form of statistical network modeling used to reverse-engineer probabilistic causality among variables; with the development of high-throughput sequencing technology, Bayesian networks have been widely used to infer causal gene regulatory networks in different diseases^62–67^. Recent studies have applied Bayesian networks to infer molecular mechanisms and key drivers in Alzheimer’s disease^24,68^. However, Bayesian networks have substantial limitations with respect to inferring opposite causality given the symmetry of joint probability. Recent work has demonstrated that bottom-up causality inference can accurately distinguish true causality from opposite causality in equivalent classes^69^. Our group has developed a computational network model, called predictive network modeling, which integrates conventional (top-down) Bayesian networks with bottom-up causality inference in order to address the problem of opposite causality inference in Bayesian network modeling. In this study, we used our causal predictive network pipeline to incorporate multi-scale omics data, including genotypes and transcriptomic profiles, in the deconvoluted neuron-specific residuals of the MAYO and ROSMAP datasets in order to build causal predictive networks separately in both datasets.

We then agnostically identified neuron-specific gene regulatory network models and key genetic drivers predicted to modulate pathological Aβ and hyperphosphorylated tau accumulation in AD. To evaluate and ensure the robustness of our results, we performed the integrative analysis and key driver identification independently in the two cohorts and cross-validated the results at every step of the analysis. In total, we reconstructed 11 causal network models combined across the two separate analyses and predicted a total of 1563 potential key drivers modulating neuronal network states and AD pathology under LOAD.

To experimentally validate our network prediction, we then prioritized 19 targets which replicated across the two cohorts. We used shRNA-mediated knockdown in human induced pluripotent stem cell (iPSC)-derived neurons^70–72^ and measured levels of Aβ38, Aβ40, and Aβ42 as well as tau and p231-tau. Among the 19 targets, we identified 10 targets which affected Aβ (*JMJD6*, *NSF*, *NUDT2*, *DCAF12*, *RBM4*, *YWHAZ*, *NDRG4*, and *STXBP1*) and/or tau/p-tau levels (*JMJD6*, *FIBP*, and *ATP1B1*). Finally, to further validate our network models and to provide insights into network connectivity, we measured the whole-genome RNA expression of the iPSC-derived neurons after knocking down each of the 19 targets and compared the differential expression (DE) signature of each target to its downstream structure in the networks. We investigated pathways enriched by the gene knockdown DE signatures to shed light on the molecular mechanisms associated with LOAD, identifying the 10 validated targets as upstream regulators of master regulatory proteins REST and VGF.

## Results

### An integrative systems biology approach for constructing single cell-type regulatory networks of AD

**Fig. 1:**
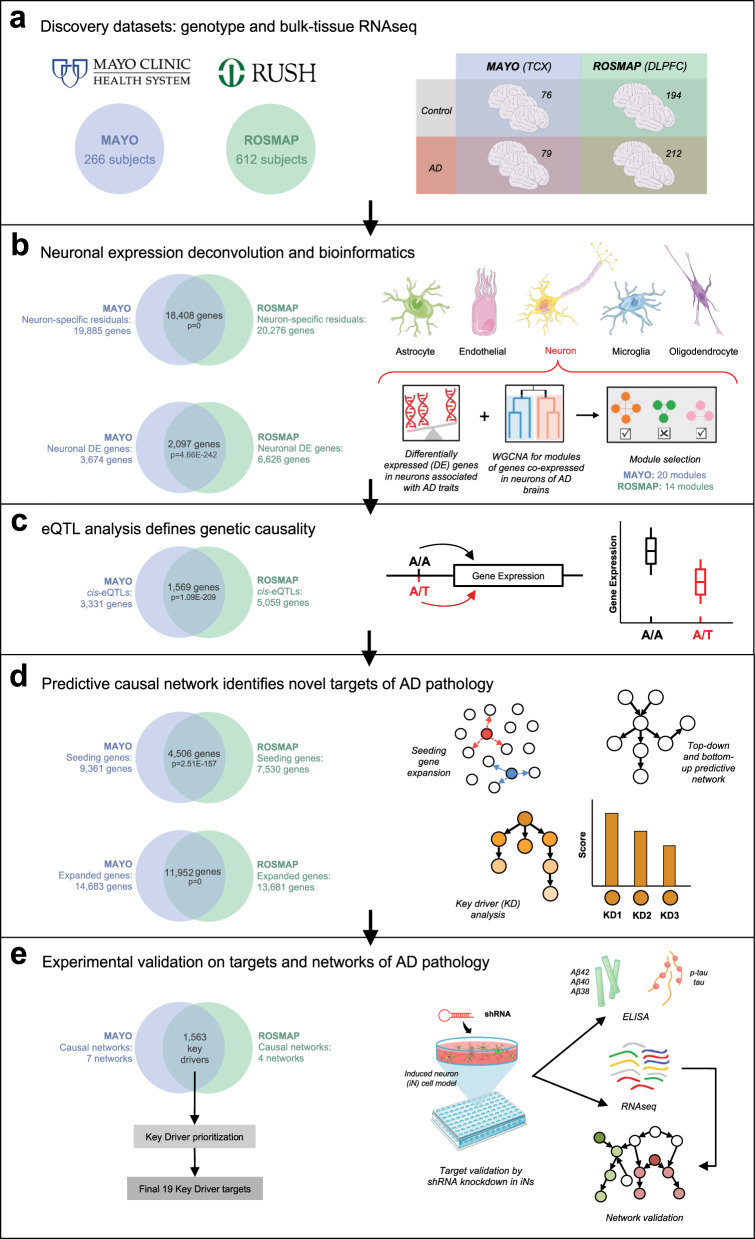
Integrative network analysis pipeline to construct data-driven neuron-specific predictive networks of AD and predict key drivers associated with AD pathology. **a** Discovery datasets include whole-genome genotype and RNAseq data of temporal cortex from the MAYO cohort and dorsolateral prefrontal cortex from the ROSMAP cohort in the AMP-AD consortium. Numbers in circles indicate the total number of subjects in each dataset with quality-controlled matched genotype and RNAseq data used in this study, whereas numbers in the table indicate the number of individuals of each phenotype used in the subsequent DE, co-expression module, and predictive network analyses. **b** Computational deconvolution of bulk-tissue RNAseq data into 5 single cell-type RNAseq sets per cohort dataset, followed by DE analysis and weighted gene co-expression network analysis in each cohort’s neuron-specific gene expression dataset. **c** mRNA expression and quantitative trait loci association analysis in each dataset provides a source of systematic perturbation for network reconstructions. **d** Construction of neuron-specific predictive network models and identification of key drivers (master regulators) from each dataset. **e** Prioritization of key driver targets from both datasets and experimental validation by shRNA-mediated gene knockdown in human iPSC-derived neurons. Venn diagrams on panels **b**–**e** indicate cross-validation at each step of the bioinformatics analyses performed independently in parallel for the MAYO and ROSMAP datasets, resulting in a single set of key driver targets. Statistical tests for each comparison are described in the text where relevant. Parts of this figure utilize graphics from Servier Medical Art (smart.servier.com) provided by Servier, licensed under a Creative Commons Attribution 3.0 Unported License. TCX temporal cortex, DLPFC dorsolateral prefrontal cortex, WGCNA weighted gene co-expression network analysis, eQTL expression quantitative trait locus. See also Supplementary Fig. 1.

We developed an integrative network analysis pipeline to construct data-driven neuron-specific predictive networks of AD (Fig. 1). The overall strategy for elucidating the single cell-type gene network model depicted in Fig. 1 centers on the objective, data-driven construction of causal network models, which can be directly queried to identify the network components causally associated with AD as well as the master regulators (key drivers) of these AD-associated components. This model also predicts the impact of the key drivers on the biological processes and pathology involved in AD, moving us towards precision molecular models of disease. We previously developed this network reconstruction algorithm, i.e., predictive network, which statistically infers causal relationships between DNA variation, gene expression, protein expression, and clinical features measured in hundreds of individuals^23,59,60^.

The inputs required for our network analysis are the molecular and clinical data generated in the MAYO and ROSMAP populations, as well as first order relationships between these data such as quantitative trait loci (QTLs) associated with the molecular traits. These relationships are input as structure priors to the network construction algorithm as a source of perturbation, boosting the power to infer causal relationships at the network level, as we and others have previously shown^21,23,24,60,73–80^. To focus on the component of AD that is intrinsically encoded in neurons, we identified the neuron-specific expression component in each cohort by applying the PSEA deconvolution algorithm^52^ to the MAYO and ROSMAP transcriptomic datasets independently (Supplementary Fig. 1, Step 1). We further focused on the molecular traits associated with AD by identifying DE gene signatures—comprised of several thousands of gene expression traits—between AD and cognitively normal samples for each dataset (Supplementary Fig. 1, Step 2). To identify correlated gene expression traits associated with AD, we constructed gene co-expression networks for each dataset, and from these networks we identified highly interconnected sets of co-regulated genes (modules) that were significantly enriched for AD gene signatures (the significant DE genes) as well as for pathways previously implicated in AD (Supplementary Fig. 1, Step 3). To obtain a final set of genes for input into the causal network construction process for each dataset, we combined genes in the co-expression network modules enriched for AD signatures (Supplementary Fig. 1, Step 5—Module selection) and performed the pathFinder algorithm^60^ to enrich the seeding gene set by including genes upstream and downstream of this set from a compiled pathway database (Supplementary Fig. 1, Step 5—Seeding expansion). We note that during the expansion of seeding genes, we only include additional genes from the compiled pathway database as nodes in the network, and we discard the disease non-specific edges so as not to bias the process of data-driven network structure learning. The edges of the final extended networks are solely inferred from AD-specific data in each cohort.

With our input set of neuron-centered genes for the AD network constructions defined, we mapped expression-QTLs (eQTLs) for neuron-specific gene expression traits in each dataset to incorporate the eQTLs as structure priors in the network reconstructions, given that they provide a systematic perturbation source that can boost the power to infer causal relationships (Supplementary Fig. 1, Step 4)^24,73,74,76–81^. The input gene set and eQTL data were then processed by an ensemble of causal network inference methods, including Bayesian networks and our recently developed top-down and bottom-up predictive networks^23,59,60,69^, in order to construct probabilistic causal network models of AD independently in the MAYO and ROSMAP cohorts (Supplementary Fig. 1, Step 6). We next applied a statistical algorithm to detect key driver genes in each given network structure^82^ and to identify and prioritize master regulators in the AD networks (Supplementary Fig. 1, Step 7). These key drivers derived from the individual networks across datasets were then pooled and prioritized based on ranking scores of impact and robustness (see ‘Methods’), resulting in a final group of 19 top-prioritized key drivers for which we performed functional validation in a human iPSC-derived neuron system. The entire analysis workflow for the independent datasets, resulting in this final group of replicated targets, is illustrated in Fig. 1.

### MAYO and ROSMAP study populations and data processing

Our causal network pipeline starts by integrating whole-genome genotyping and RNAseq data generated from patients spanning the complete spectrum of clinical and neuropathological traits in AD. We used patient data from two separate cohorts within the AMP-AD consortium: temporal cortex data from 266 subjects in MAYO^83–85^ and dorsolateral prefrontal cortex data from 612 subjects in ROSMAP^22,86–88^ (Fig. 1a). We processed matched genotype and RNAseq data separately in each dataset (Fig. 1, Supplementary Fig. 1; see ‘Methods’).

Central nervous system (CNS) tissue consists of various cell types, including neurons, glia, and endothelial cells. To discover key network drivers specific to a single cell type in the CNS and study their contribution to AD in that specific cell type, we utilized verified single-cell marker genes to directly deconvolve bulk-tissue gene expression data into cell type-specific gene expression for the five major cell types in the CNS: neurons, microglia, astrocytes, endothelial cells, and oligodendrocytes (see ‘Methods’). In this study, we focused on investigating the role of neuronal cells in AD, as they are the primary cell type affected by AD pathogenesis^89–94^. After normalizing the bulk-tissue RNAseq data, we performed variance partition analysis (VPA)^95^ to evaluate the contributions of cell type-specific markers as well as demographic, clinical, and technical covariates (such as batch effects) to the gene expression variance before performing any covariate adjustment (Supplementary Fig. 2a, b). The cell type-specific marker genes used for neurons, microglia, astrocytes, endothelial cells, and oligodendrocytes were *ENO2*^96^, *CD68*^97^, *GFAP*^98^*, CD34*^99^, and *OLIG2*^100–102^, respectively, as previously published having been obtained directly under the AD condition at the protein level. The VPA results reflect the prominent effect of CNS cell types on the variance of the brain RNAseq data. In the MAYO dataset, the additional covariates used in the VPA included exonic mapping rate, RNA integrity number, sequencing batch, diagnosis, age at death, tissue source, *APOE* genotype, and sex. In the ROSMAP dataset, we were able to include the same covariates with the exception of tissue source and the addition of age at first AD diagnosis, post-mortem interval, education, and study (ROS or MAP).

We then performed covariate adjustment and deconvolution using the PSEA method^52^ in each dataset, calculating gene expression residuals using a linear regression model to adjust the normalized bulk-tissue expression data with demographical and technical covariates as well as the cell type-specific markers. Cell type-specific gene expression, including the neuron-specific component, was directly derived by adding the estimated variance of each cell type to the residual (see ‘Methods’), avoiding the need to first estimate the cell population from bulk tissue data, which could induce approximation errors. We then repeated VPA in the neuron-specific residuals of each dataset to demonstrate that our deconvolution and covariate adjustment methods properly capture the neuronal component while removing potential confounds such as batch effect, age, and sex (Supplementary Fig. 2c, d). Finally, to justify the use of single cell type-specific markers for deconvolution by the PSEA method, we performed a set of analyses comparing multiple cell type-specific biomarker lists (derived from existing scRNAseq studies) to each other (Supplementary Fig. 3a), to our AD residuals (Supplementary Fig. 3b, c), and to the AMP-AD Agora list of potential therapeutic targets in AD (Supplementary Fig. 3j, k), as well as a robustness analysis demonstrating that our neuron-specific residual derived from *ENO2* expression represents a robust neuronal component in the bulk-tissue RNAseq data when compared to random selections of multi-gene neuronal biomarkers derived from these scRNAseq datasets in AD (Supplementary Fig. 3b–i; see ‘Methods’).

### Identifying AD-associated gene signatures in neurons and mapping their eQTLs

**Fig. 2:**
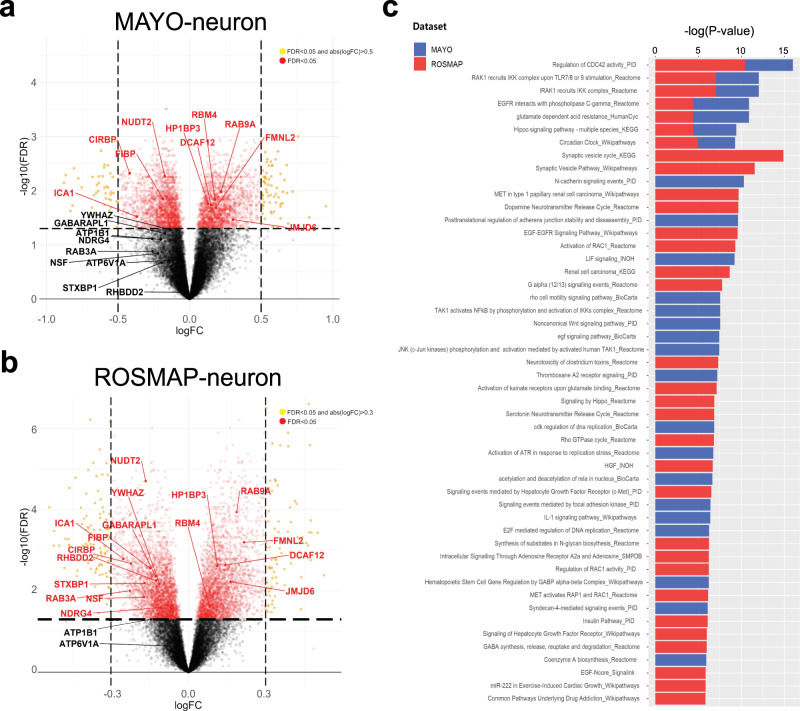
Neuron-specific gene expression signatures in AD. DE analysis of deconvoluted neuron-specific residuals identifies a robust DE signature associated with the difference between AD patients and cognitively normal controls. **a**, **b** All significantly up- and down-regulated genes for MAYO-neuron (**a**) and ROSMAP-neuron (**b**). Significance was assessed by t-test with FDR < 0.05; thresholds of logFC shown in each volcano plot are for visualization only. MAYO (*n* = 79 and 76 for AD and cognitively normal) and ROSMAP (*n* = 212 and 194 for AD and cognitively normal). Gene symbols are highlighted in red text for the 19 key drivers later prioritized for experimental validation in vitro. See also Supplementary Fig. 4. **c** Pathway enrichment analysis on the neuron-specific DE expression signatures using human ConsensusPathDB reveals dysregulated biological processes associated with AD. Significance was assessed by Fisher’s exact test with *p*-value < 0.05. Source data for this panel is provided in Supplementary Data 13. Detailed statistical results of DE genes and enriched pathways are summarized in Supplementary Data 1 and 2, respectively, and overlapped pathways are summarized in Supplementary Data 13.

To identify an AD-centered set of neuronal gene expression traits, we performed DE analysis using the deconvoluted neuron-specific expression residuals in the MAYO and ROSMAP cohorts (see ‘Methods’). In comparing expression data between AD and cognitively normal controls (MAYO: 79 AD, 76 control; ROSMAP: 212 AD, 194 control), there were 3674 significant DE neuron-specific genes in the MAYO dataset (hereby MAYO-neuron) and 6626 neuron-specific DE genes in the ROSMAP dataset (hereby ROSMAP-neuron) (Fig. 2a, b; Supplementary Fig. 4; Supplementary Data 1). There were 2097 significant DE genes overlapping between the two datasets (Fisher’s exact test, odds ratio = 3.9784, *p*-value = 4.66E-242), thus cross-validating the neuron-specific DE signatures independently derived from the two cohorts.

To examine the biological processes that are dysregulated in AD cases versus controls as reflected in the DE signatures, we performed pathway enrichment analysis on the MAYO-neuron and ROSMAP-neuron gene sets using Human ConsensusPathDB^103–107^. We identified 75 and 73 enriched pathways in each dataset, respectively, with 7 pathways that were significantly dysregulated in both datasets (Fig. 2c; Supplementary Data 2, 13). These signatures were enriched for a number of cellular/molecular pathways, including those involving CDC42^108–110^, IRAK/IKK^111–113^, EGFR/PLCG^114^, GAD^115^, Hippo^116^, and clock genes^117,118^, some of which have been implicated and/or interrogated in AD previously. Additional pathways of note implicated by a single cohort dataset with known relevance to amyloid and/or tau pathology include those related to NF-κB activation^119,120^ and N-cadherin signaling^121,122^.

We further validated our neuron-specific DE signatures in AD, which were derived from deconvoluted bulk-tissue RNAseq data, by comparing our MAYO-neuron and ROSMAP-neuron DE genes with the excitatory and inhibitory neuronal signatures identified by a separate study that generated scRNAseq data from the same ROSMAP cohort^123^. We employed the sampling-based method described in ref. ^123^ and first compared the pair-wise enrichment among the scRNAseq-derived DE gene signatures in the ROSMAP dataset^123^ for excitatory neurons, inhibitory neurons, astrocytes, oligodendrocytes, oligodendrocyte progenitor cells, and microglial cells (Supplementary Data 3). Briefly, we found that the excitatory neuron signature significantly overlaps with inhibitory neurons, astrocytes, oligodendrocytes, and oligodendrocyte progenitor cells (FDR = 1.24E-25, 2.26E-03, 2.69E-04, and 1.36E-02, respectively) but is not enriched for microglia (FDR = 0.58). We also found that the inhibitory neuron signature is significantly enriched for astrocytes (FDR = 3.83E-02) and oligodendrocytes (FDR = 9.30E-07), but not oligodendrocyte progenitor cells (FDR = 1) or microglia (FDR = 1). The significant overlap among scRNAseq-derived DE signatures of different cell types highlights the intrinsic biological interactions among different cell types in the AD brain. Next, we found a similar pattern of enrichment between our MAYO-neuron and ROSMAP-neuron DE signatures and scRNAseq-derived DE gene signatures from ROSMAP^123^ (Supplementary Data 4), i.e. their excitatory neuron signature (FDR = 3.41E-10 and 3.56E-17, respectively) as well as their inhibitory neuron signature (FDR = 0.0285 and 0.00429, respectively), demonstrating significant correlation between our deconvoluted neuron-specific DE signatures and scRNAseq-derived neuronal signatures in AD. The greater excitatory-neuronal enrichment among our deconvoluted neuron-specific DE signatures is consistent with ref. ^123^ and similarly suggests that our deconvoluted RNAseq datasets capture the aberrant increases in neuronal excitotoxicity associated with AD in humans^124^. Thus, overall, despite the inherent multi-cell type interactions revealed by these analyses, we argue that our deconvoluted neuron-specific AD signatures are robust and provide a complementary solution to single cell-type transcriptomics analysis.

Another critical input for the construction of Bayesian network and causal predictive network models are the eQTLs, leveraged as a systematic source of perturbation to enhance causal inference among molecular traits. This is an approach we and others have demonstrated across a broad range of diseases and data types^24,69,73–77,79–81,125–138^. We mapped *cis*-eQTLs by examining the association of neuron-specific expression traits with genome-wide genotypes^18,139–141^ assayed in the MAYO and ROSMAP cohorts (see ‘Methods’). In the MAYO- and ROSMAP-neuron sets, 3331 (16.8%) and 5059 (25.0%), respectively, of the residual genes were significantly correlated with allele dosage (FDR < 0.01) (Supplementary Data 5). Of the *cis*-eQTLs detected in each cohort, 1569 genes were overlapping between the two sets (47% of MAYO *cis*-eQTLs and 31% of ROSMAP *cis*-eQTLs, Fisher’s exact test, *p*-value = 1.09E-209), providing further validation of the two independent cohorts.

### Neuronal co-expression networks associated with LOAD

**Fig. 3:**
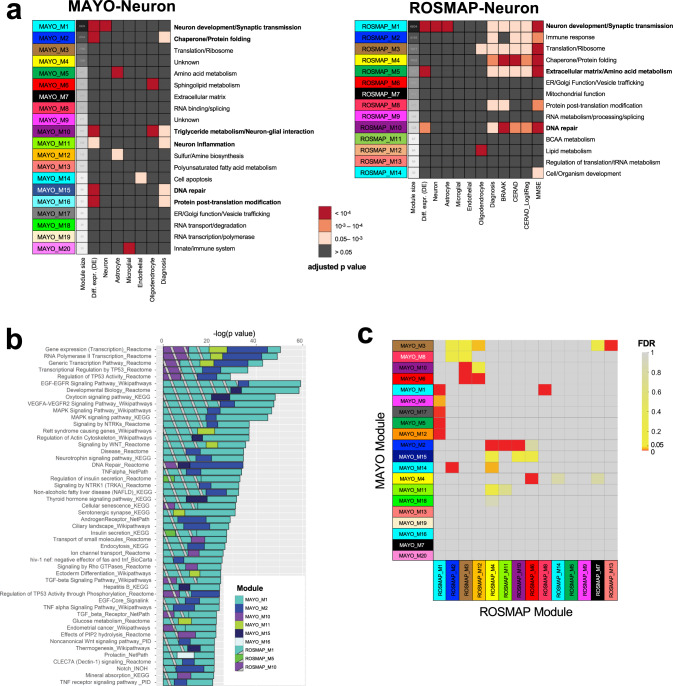
Neuron-specific co-expression analysis identifies robust gene modules enriched for biological processes associated with AD. **a** Neuron-specific co-expression network analyses in the MAYO and ROSMAP cohorts identify gene modules associated with AD in each dataset. Module functions for each dataset were characterized by significantly enriched biological processes, with bold text indicating neuron-specific modules selected for further analysis. Each module was evaluated based on enrichment for neuron-specific DE genes, for scRNAseq derived neuron-specific biomarker genes, and for categories of available AD traits (MAYO and ROSMAP diagnosis by ANOVA; BRAAK, CERAD, and MMSE by linear regression). We also evaluated enrichment for scRNAseq-derived biomarker genes for microglia, astrocytes, endothelial cells, and oligodendrocytes to cross-validate that modules enriched for neuron-DE genes were not enriched for other cell types. Significance was assessed by Fisher’s exact test with FDR < 0.05 (*n* = 30 to 6929 for MAYO and 34 to 6604 for ROSMAP). **b** Among the neuron-specific modules in the two datasets, pathway enrichment analysis identifies robust pathways associated with AD which replicate in both cohorts. Source data is provided in Supplementary Data 2. **c** Cross-validation of neuron-specific co-expression modules between MAYO and ROSMAP identifies pairs of modules with significantly overlapping gene members. Significance was assessed by Fisher’s exact test with FDR < 0.05. See Supplementary Data 6 for further information on the biological processes significantly enriched in each module.

While DE analysis can reveal patterns of neuron-specific expression associated with AD, the power of such analysis to detect a small-to-moderate expression difference is low. To complement the DE analyses in identifying the input gene set for the causal network, we clustered the neuronal gene expression traits into data-driven, coherent biological pathways by constructing co-expression networks, which have enhanced power to identify co-regulated sets of genes (modules) that are likely to be involved in common biological processes under LOAD. We constructed co-expression networks on the AD patients within each dataset after filtering out lowly expressed genes (see ‘Methods’), resulting in the MAYO-neuron co-expression network consisting of 20 modules ranging in size from 30 to 6929 gene members and the ROSMAP-neuron co-expression network consisting of 14 modules ranging from 34 to 6604 gene members (Fig. 3a).

To evaluate the functional relevance of each cohort’s neuron-specific modules to AD pathology, we performed enrichment analysis of each module for its AD-associated neuronal DE signatures, known single-cell marker genes for the 5 major cell types in the CNS^57^, and categories of AD traits available from its respective cohort (Fig. 3a). From these enrichment results, we identified neuron-specific modules associated with AD DE genes: M1, M2, M10, M11, M15, and M16 from MAYO and M1, M5, and M10 from ROSMAP. It is worth noting that similar to the DE signatures (Supplementary Data 3), a small number of the MAYO- and ROSMAP-neuron co-expression modules are significantly enriched for astrocyte and oligodendrocyte biomarkers, highlighting the intrinsic cellular interactions between these cell types in the AD brain.

To further characterize the biological processes involved in the co-expression modules from each dataset, we performed pathway enrichment analysis to identify overrepresented biological processes within and across the modules (Fig. 3b; Supplementary Data 6). Out of the selected AD-associated modules from the MAYO- and ROSMAP-neuron co-expression networks, respectively, we found 36 and 16 significantly enriched pathways based on the Human ConsensusPathDB database, with 11 enriched pathways overlapping between the two datasets (Fisher’s exact test, odds ratio = 383.87, *p*-value = 5.41E-21; Supplementary Data 14). In comparing all pairs of modules between the datasets, we identified 17 module pairs with significant overlap of gene members (Fig. 3c), demonstrating the robustness of the two independent co-expression networks.

### Ensemble of neuron-specific, causal gene regulatory networks identifies pathological pathways and key drivers for neuronal function in AD

The ultimate goal of this study was to identify upstream master regulators (key drivers) of neuronal pathways that contribute to AD. Following our DE, eQTL, and co-expression network analyses, we built an ensemble of causal network models—including standard Bayesian networks^22,24^ and state-of-the-art predictive network models^21,23,60^—by integrating the eQTLs and deconvoluted neuron-specific RNAseq residuals.

We first pooled all genes from the selected AD-associated modules per dataset (six MAYO-neuron modules and three ROSMAP-neuron modules, indicated in Fig. 3a) to create a seeding set of genes for each cohort for input into the network models. This resulted in 9361 seeding genes from the MAYO-neuron co-expression network and 7530 seeding genes from the ROSMAP-neuron co-expression network. We note an overlap of 4506 genes between the two seeding gene sets (48.1% of MAYO and 59.8% of ROSMAP, Fisher’s exact test, odds ratio = 2.875, *p*-value = 2.51E-157), indicating the reproducibility of these analyses across the two independent datasets. To further improve the robustness of our network models, we also expanded each set of seeding genes by including their known upstream and downstream genes in each cohort’s co-expression network, extracted from signaling pathway databases using the pathFinder algorithm^60^ (see ‘Methods’; note that we did not include the gene-gene interactions as prior edge information for network construction). Co-expression network modules are only sensitive to linear relationships between pairs of genes, whereas non-linear gene regulations will not be captured by co-expression analysis. This expansion step thus includes genes in the same pathways as the seeding genes which otherwise failed to be included in the same module derived from the co-expression networks, resulting in 14,683 expanded genes from MAYO-neuron, 13,681 expanded genes from ROSMAP-neuron, and an overlap of 11,952 genes between the two expanded gene sets (Fisher’s exact test, *p*-value = 0). The use of both the seeding gene set and the expanded gene set for analysis of the MAYO and ROSMAP datasets therefore increases the power to build robust networks and to discover high-confidence neuronal key drivers associated with AD pathology.

We also incorporated *cis*-eQTL genes into each network as structural priors. As *cis*-eQTLs causally affect the expression levels of neighboring genes, they can serve as a source of systematic perturbation to infer causal relationships among genes^23,59,60,81^. Of the 3331 and 5059 unique *cis*-eQTL genes identified in the MAYO- and ROSMAP-neuron datasets, respectively, 687 and 1978 overlapped with the seeding gene set and 2,162 and 2,998 overlapped with the expanded gene set. We finally proceeded to build Bayesian networks and predictive networks using the two sets of genes per dataset—i.e., 9361 seeding and 14,683 expanded genes for the MAYO dataset and 7530 seeding and 13,681 expanded genes for the ROSMAP dataset—and incorporating each dataset’s *cis*-eQTL genes as structural priors.

**Fig. 4:**
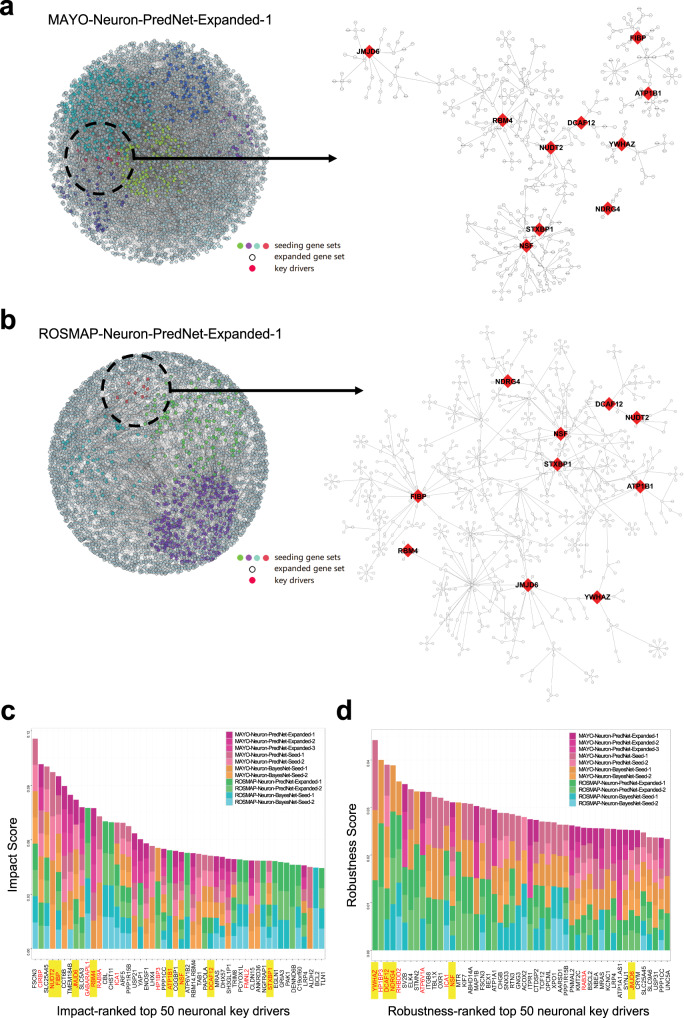
Neuron-specific causal network analyses identify molecular mechanisms and key driver targets associated with AD. **a**, **b** Two predictive networks out of the final 11 neuron-specific causal Bayesian and predictive network models derived from the MAYO (**a**) and ROSMAP (**b**) seeding and expanded gene sets. The MAYO and ROSMAP networks shown here were built from their respective expanded gene sets with posterior probability cut-offs of 0.5 and 0.3, respectively. Close-up views show the 10 key driver targets which were validated in vitro (red) along with their neighboring downstream subnetworks. See also Supplementary Fig. 5. **c**, **d** The top 50 out of 1563 total key driver targets ranked individually according to impact (**c**) and robustness (**d**) scores across the 11 independent MAYO-neuron and ROSMAP-neuron Bayesian and predictive networks. Red text indicates prioritized key drivers; yellow highlights those which were validated in vitro. Source data is provided in Supplementary Data 15.

Since structure learning is a heuristic and stochastic process, we applied a wide range of cut-offs on the posterior probability of edges to derive sets of robust Bayesian and predictive network structures for each dataset. For the MAYO-neuron seeding gene set, we built Bayesian networks and applied two posterior probability cut-offs (0.4 and 0.5, see ‘Methods’) to get two MAYO-neuron Bayesian networks (MAYO-Neuron-BayesNet-Seed-1 and -2) which were comprised of 9111 and 9044 genes, respectively. In addition, we built predictive networks with the same two posterior probability cut-offs (0.4 and 0.5) to derive two MAYO-neuron predictive networks (MAYO-Neuron-PredNet-Seed-1 and -2), which also included 9111 and 9044 genes, respectively. For the MAYO-neuron expanded gene set, we built predictive networks and chose three posterior probability cut-offs (0.5, 0.6, 0.7) to get three MAYO-neuron predictive network models (MAYO-Neuron-PredNet-Expanded-1, -2, and -3), which were comprised of 14,238, 13,926, and 13,365 genes, respectively. For the ROSMAP-neuron seeding gene set, we built Bayesian networks and applied two cut-offs (0.3 and 0.4) to derive two Bayesian networks (ROSMAP-Neuron-BayesNet-Seed-1 and -2) which consisted of 6786 and 6756 genes, respectively. For the ROSMAP-neuron expanded gene set, we built two predictive networks and chose two cut-offs (0.3 and 0.4) to build two predictive networks (ROSMAP-Neuron-PredNet-Expanded-1 and -2) consisting of 12,147 and 12,074 genes, respectively. Thus, in total from the MAYO and ROSMAP datasets, we derived 11 networks for the inference of a robust set of key drivers, using several different network reconstruction methods, network gene sets, and posterior cut-offs. We demonstrate 2 of the final 11 causal network models in Fig. 4a, b (MAYO-Neuron-PredNet-Expanded-1 and ROSMAP-Neuron-PredNet-Expanded-1), and the remaining 9 causal networks are shown in Supplementary Fig. 5.

### Identification and prioritization of neuronal key drivers regulating AD pathology

Having generated the causal predictive networks from the MAYO-neuron and ROSMAP-neuron datasets, we applied key driver analysis^82^ to derive a list of key driver genes from each network. Key driver analysis seeks to identify genes in a causal network which modulate network states; in the present analysis, we applied this analysis to identify genes causally modulating the network states of our neuron-specific Bayesian and predictive network models. In total, we identified 1563 key driver genes across the 11 independent networks.

To prioritize key drivers for further investigation, we first ranked the 1563 initial key driver targets according to two separate measures: an impact score and a robustness score (see ‘Methods’). Briefly, the impact score is a predicted value quantifying the regulatory impact of a given key driver on its downstream effector genes associated with AD pathology. Intuitively, the shorter a path from a key driver to its downstream effectors in a network—with less other parental co-regulators along the same path—the greater the impact of this target on its effectors in that network. The robustness score is reflective of the number of datasets (MAYO and/or ROSMAP), gene sets (seeding and/or expanded), and network models (Bayesian and/or predictive) by which a key driver is replicated. After ranking the total 1563 neuron key drivers according to each score, we focused on the top 50 key drivers in each ranked list (Fig. 4c, d; Supplementary Data 15).

We then performed a series of steps to prioritize a final group of key driver targets for in vitro experimentation out of the ensemble of the top 50 ranked candidates for each score. We first calculated the replication frequency across the two ranked lists and identified 11 replicated targets, indicating robustness across these two independent ranking scores, and 39 unique targets in each ranked list (78 total). For the 11 replicated targets, we removed any which ranked lower than 15 in both scores, resulting in 7 top-ranked targets (*ICA1*, *NSF*, *FSCN3*, *HP1BP3*, *DCAF12*, *JMJD6*, and *SLC25A45*) which were replicated in both lists and ranked within the top 15 in one or both scores. Next, for the remaining 78 unique targets, we first selected the top 3 unique targets from each ranked list (*CIRBP*, *NUDT2*, and *FIBP* for impact score; *YWHAZ*, *NDRG4*, and *RHBDD2* for robustness score). To further select targets from the remaining 36 neuron-specific targets in each ranked list (72 total), we identified 4 targets (*GABARAPL1, ATP1B1, ATP6V1A*, and *RAB3A*) which were previously nominated to the AMP-AD Agora list based on separate data-driven network analysis using the bulk-tissue RNAseq data in the MAYO and ROSMAP datasets with the same approach as this study^142^. Finally, to balance our selection strategy, we selected an additional 4 targets (*RBM4*, *RAB9A*, *FMNL2*, and *STXBP1*) out of the lower-to-middle ranked top 50 unique targets based on the availability of proper constructs.

In summary, we prioritized a group of 19 targets for experimental validation in vitro (Fig. 4c, d, highlighted in red) by selecting the top-ranked replicated targets across the two scores (we note that *SLC25A45* and *FSCN3* were excluded at this stage due to lack of proper constructs), 6 top-ranked unique targets (top 3 from each score), 4 targets overlapping with prior data-driven nominations to the AMP-AD Agora list, and 4 lower-to-middle ranked targets.

### Validation of AD-associated function of neuronal key drivers by knockdown in human neurons

**Fig. 5:**
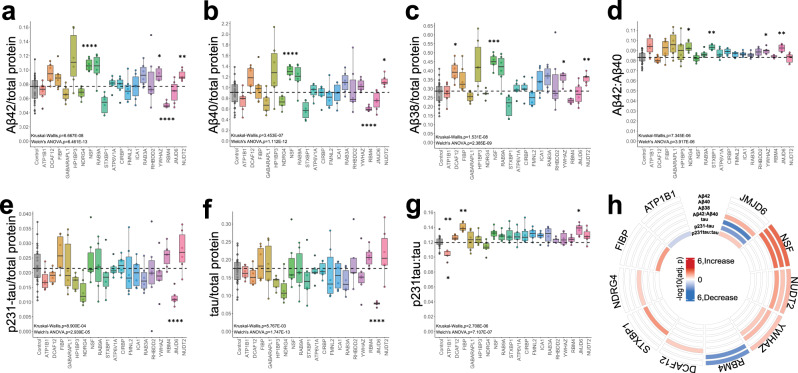
Human iPSC-derived neurons show altered Aβ species and tau/phospho-tau levels following shRNA-mediated knockdown of selected target genes. **a**–**c** Secretion of Aβ42 (**a**), Aβ40 (**b**), and Aβ38 (**c**) was measured in conditioned media by ELISA, normalized to the average of controls (no transduction and empty vector) as well as to total protein in the neuronal cell lysate. The ratio of Aβ40:42 was also calculated (**d**). **e**, **f** p231-tau (**e**) and total tau (**f**) were measured in cell lysates by ELISA, normalized to the average of controls (no transduction and empty vector) as well as to total protein in the cell lysate. The ratio of p231-tau to total tau was also calculated (**g**). For all panels, a black dashed line indicates the median for control conditions for that measurement. For each gene knockdown condition, 3 shRNA constructs were used, each shRNA construct was used in duplicate wells, and each dot represents data from one well. For each boxplot, the box contains the middle quartiles of the data and black bar denotes the median value, and the upper and lower quartiles contain the maximum and minimum values and the remaining 50% of the data. Source data is provided in Supplementary Data 16. For each measured parameter, we first performed a Welch’s ANOVA with unequal variance to detect significant differences across conditions, with an additional non-parametric Kruskal-Wallis ANOVA to confirm the significance; *p*-values are indicated on each panel. We then used a Dunnett’s T3 multiple comparisons test to compare each target shRNA to the control condition for each parameter; *adj-*p* < 0.05, **0.001 < adj-*p* < 0.05, ***0.0001 < adj-*p* < 0.001, ****adj-*p* < 0.0001. (*n* = 5–29). **h** Circus plot summarizing the effects of the 10 key driver targets found to modulate levels of Aβ42, 40, 38, Aβ42:40, tau, p231-tau and/or p231-tau:tau. Significance was assessed by -log10(Dunnett’s T3 adjusted *p*-value). Red indicates that knockdown of the target significantly increased the given measurement value, whereas blue indicates that knockdown significantly decreased the value. Data frequency distributions and detailed statistical results for this figure are provided in Supplementary Fig. 6 and Supplementary Data 7, respectively.

We next aimed to test the functional consequences of perturbation of the top candidate driver genes in human neurons. Healthy control human iPSCs were differentiated to a neuronal fate using a modified version of the well-established NGN2 differentiation protocol^71^, which rapidly generates induced neurons (iNs) which are most similar to layer 2/3 glutamatergic neurons of the cerebral cortext^71,72,143^. By 2 weeks in culture, iNs are post-mitotic, electrically active, and express a full array of synaptic markers^71,143^. In order to perturb the expression of the top 19 candidate key driver genes, we obtained sets of validated short hairpin RNA (shRNA) constructs packaged in lentivirus, with each set containing three constructs against each selected gene. At day 17 of differentiation, iNs were transduced with lentivirus encoding a single shRNA, alongside control cells which either received empty virus or were not transduced. Media were exchanged on all cells 18 h later. Five days following transduction (day 22 of differentiation), conditioned media were collected, and cells were lysed either to collect RNA for RNAseq or to harvest protein for analyses of Aβ and p-tau/tau, similar to our previous study of LOAD genome-wide association study hits^144^. All Aβ and tau data were normalized to total protein in the cell lysate per well, and all data for each shRNA knockdown were additionally normalized to the average of control conditions (empty vector and no transduction) (Fig. 5a–g; Supplementary Data 16).

Aβ38, 40, and 42 levels were measured in conditioned media from the transduced and control iNs using the Meso Scale Discovery Triplex ELISA platform. Of the 19 genes tested, knockdown of 11 genes had no significant effect on the levels of any Aβ peptides measured nor the ratio of Aβ42 to Aβ40 (Fig. 5a–d). However, targeted knockdown of *YWHAZ* significantly raised Aβ42 peptide levels, knockdown of *DCAF12* and *YWHAZ* increased Aβ38 levels, and knockdown of *NSF* and *NUDT2* significantly increased levels of all three Aβ peptides measured (Aβ38, 40, and 42) (Fig. 5a–c). On the other hand, knockdown of *RBM4* significantly reduced levels of both Aβ42 and Aβ40 (Fig. 5a, b). Lastly, knockdown of *NDRG4*, *STXBP1*, *YWHAZ*, and *JMJD6* resulted in a significant elevation of the putatively neurotoxic Aβ42 to 40 ratio^145,146^(Fig. 5d).

We also examined levels of tau species in the transduced and control iN lysates using a Meso Scale Discovery ELISA measuring both total tau and phospho-tau (Thr231). Knockdown of 16 of the 19 candidate genes tested had no significant effect on the levels of tau, p231-tau, or the neurotoxic ratio of p231-tau to tau (Fig. 5e–g). However, targeted knockdown of *JMJD6* significantly decreased the levels of both p231-tau and tau (Fig. 5e, f). We also note that knockdown of *NSF* approached significance of increased levels of p231-tau (Fig. 5e; Dunnett’s T3 adjusted *p*-value = 0.075). Finally, knockdown of *FIBP* and *JMJD6* resulted in significant elevation of the p231-tau to tau ratio, while knockdown of *ATP1B1* significantly lowered this ratio (Fig. 5g).

Thus, we confirm modulation of AD endophenotypes in human iNs following independent reduction of the expression of 10 different genes out of the top 19 predicted key driver targets (Fig. 5h). Data frequency distributions and detailed statistical results of Aβ and tau measurements are included in Supplementary Fig. 6 and Supplementary Data 7. We additionally analyzed the overlap of these 10 targets with our DE and *cis*-eQTL analyses in MAYO and ROSMAP (Supplementary Data 8 and 9, respectively). As not all of the targets are significant DE or *cis*-eQTL genes, we conclude that our network analysis adds a critical value to the identification and prioritization of targets which cannot be achieved by DE and eQTL analyses alone.

### Validation of AD-associated networks and pathways by RNAseq of human neurons following targeted gene knockdown

**Fig. 6:**
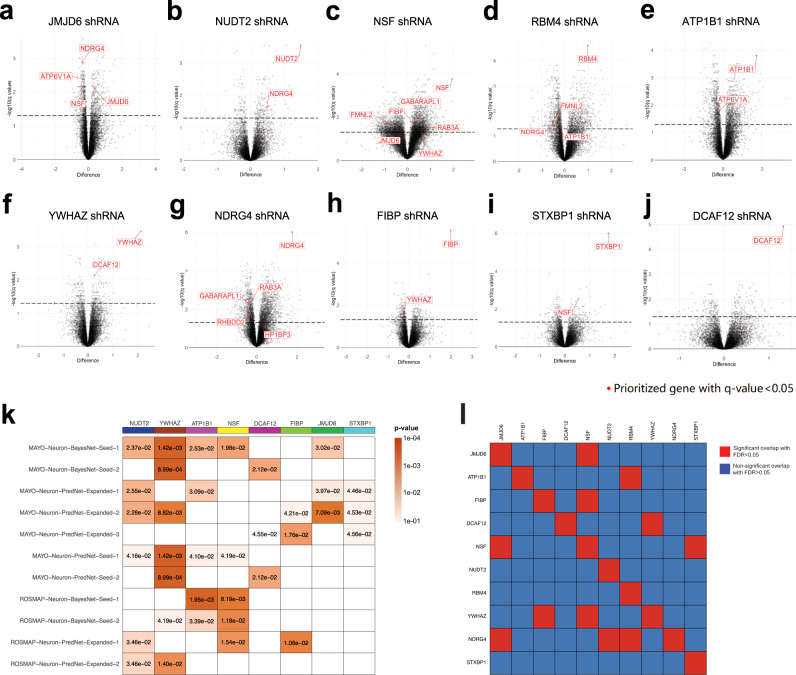
Gene expression changes following knockdown of the 10 validated targets in human iPSC-derived neurons. **a**–**j** RNAseq analysis showing significantly up- and down-regulated DE genes after shRNA-mediated knockdown of each of the 10 validated targets. Significance was assessed using the two-stage linear step-up procedure of Benjamini, Krieger, and Yekutieli with *q*-value < 0.05, indicated by the black dashed line. Red gene symbols indicate any of the prioritized 19 target genes that were significantly affected. **k** Network validation by enrichment analysis of significant DE genes following shRNA knockdown of the 10 validated targets in the 11 subnetwork networks. We compared the DE genes after knockdown of each target to the individual downstream subnetwork of that target extracted from the 11 reconstructed networks. Significance was assessed by Fisher’s exact test with *p* < 0.05. **l** Significant overlap in DE genes resulting from knockdown of each of the 10 validated targets in human iPSC-derived neurons. Significance was assessed by Kruskal-Wallis ANOVA with Dunnett’s T3 multiple comparisons test with FDR < 0.05. Detailed results are summarized in Supplementary Data 10.

To validate the network structure, we repeated shRNA-mediated knockdown of each of the 19 target key drivers in another set of cultured control iNs and subsequently measured gene expression by RNAseq. For each of the 10 AD endophenotype-modulating targets, we derived a DE signature from the RNAseq data (Fig. 6a–j, Supplementary Data 10). Next, we extracted the downstream (sub)network of each of those 10 targets from the MAYO- and ROSMAP-neuron networks and evaluated the enrichment of the knockdown DE signature by the downstream subnetworks for each target. We found that 8 out of the 10 DE signatures were enriched by the downstream subnetworks of their corresponding target (Fig. 6k), validating that our network models capture a large portion of molecular processes and pathways at the neuron level.

We then further examined the gene expression changes resulting from knockdown of the 10 validated targets. Following *JMJD6* knockdown, which significantly altered ratios of both Aβ and tau in iNs, 656 genes were significantly upregulated and 419 genes significantly downregulated (Fig. 6a). Interestingly, among those significantly upregulated genes were 3 of our other 19 key driver candidates (*NDRG4*, *ATP6V1A*, and *NSF*), indicating that their expression is affected by the reduction of *JMJD6* in neurons. Volcano plots in Fig. 6b–j highlight additional key driver candidates whose expression was affected by knockdown of each of the 9 other validated targets. Moreover, we found certain common genes affected by the perturbation of multiple validated targets: 6 genes (*FGF11*, *GIT2*, *KLHL28*, *PLCB3*, *SEPSECS*, and *SLC48A1*) were affected by knockdown of *NDRG4*, *STXBP1*, *YWHAZ*, and *JMJD6*, and 9 genes (*SEPTIN3*, *ABR*, *AOC2*, *CTFIP2*, *ZGTF2H1*, *MRPL17*, *NIIPSNAP1*, *RIMS4*, and *TMEM246*) were affected by perturbation of *DCAF12*, *NSF*, and *NUDT2*. This observation indicates that there may be unique and common molecular pathways among these validated AD endophenotype-modulating targets; we illustrate the significant overlap of DE genes after each target knockdown in Fig. 6l.

**Fig. 7:**
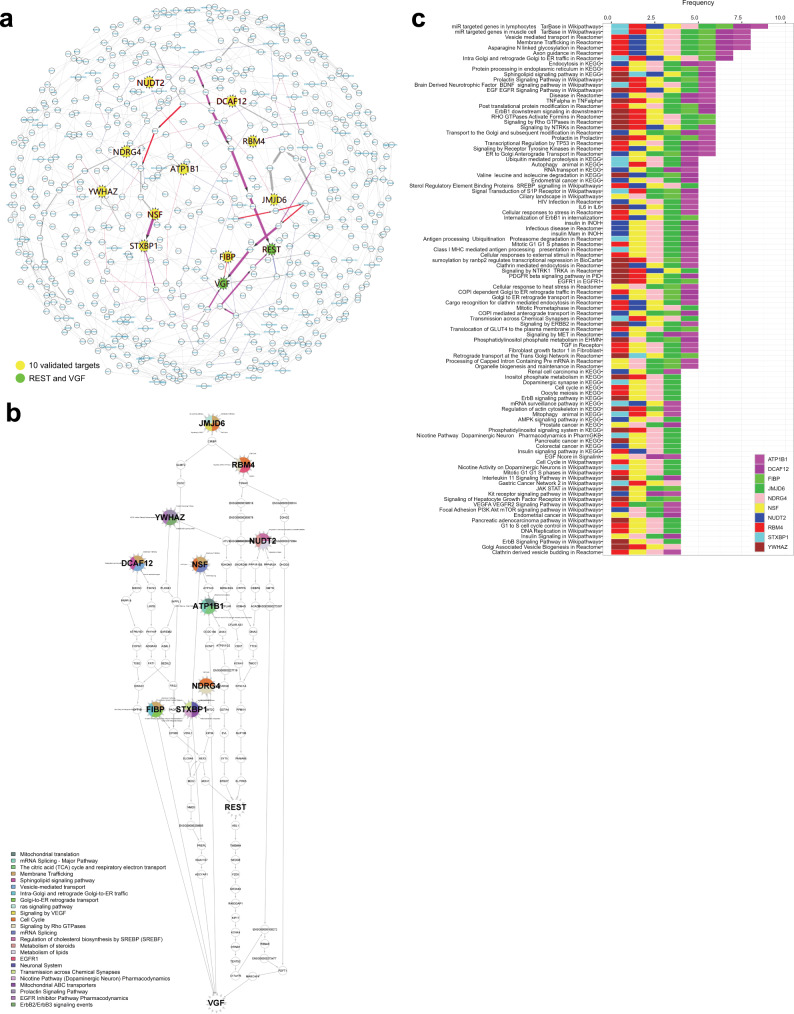
Regulatory pathway analysis reveals unique and shared biological pathways between the validated targets in each network. **a** The downstream members of each validated target were extracted from every network model, and edges from each downstream sub-network were pooled together into a consensus subnetwork of the 10 validated targets (yellow nodes). The 10 targets tightly regulate each other and are upstream regulators of both REST and VGF (green nodes). Blue, red, and purple edges, respectively, indicate the shortest paths from any of the 10 validated targets to REST, VGF, or both. Edge thickness indicates the frequency of corresponding edges appearing across all the networks. **b** The shortest paths from each of the 10 targets to REST and VGF were extracted from each network and pooled together into a hierarchical structure. The coloring of each target node annotates its representative ConsensusPathDB pathways enriched by significant DE genes in the shRNA knockdown experiments. **c** The overall ConsensusPathDB pathways significantly enriched by each of the 10 target genes (assessed by Fisher’s exact test with *p*-value < 0.05) were pooled and ranked in descending order by the frequency of enrichment by any of the targets. Detailed statistical results and descriptions of all pathways affected by these targets are provided in Supplementary Data 11.

To investigate possible mechanisms underlying these observations, we extracted regulatory pathways among the 10 validated targets in each of the 11 MAYO- and ROSMAP-neuron networks. We found that these 10 targets tightly regulate each other, and, interestingly, are all upstream regulators of the prominent proteins REST and VGF (Fig. 7a, b). REST (restrictive element 1-silencing transcription factor) is a known master regulator of neurogenesis via epigenetic mechanisms, apoptosis, and oxidative stress;^147,148^ VGF (nerve growth factor inducible) is a recently identified AD target whose overexpression in a mouse model reversed AD phenotypes^68^. In particular, our networks identified *FIBP* as a direct upstream regulator of VGF. Our findings thus indicate that these 10 targets may modulate AD-related pathology partially through REST and VGF pathways.

Finally, we performed pathway enrichment analysis (see ‘Methods’) on the DE signatures derived from the RNAseq data in order to identify the unique and shared pathways affected by the knockdown of the 10 AD endophenotype-modulating targets (*JMJD6*, *NSF*, *NUDT2*, *DCAF12*, *RBM4*, *YWHAZ*, *NDRG4*, *STXBP1*, *FIBP* and *ATP1B1*). We compared significant pathways enriched by the DE signature of each of the targets and found that 1 pathway is shared by 9/10 targets, 4 pathways are shared by 8/10 targets, 2 pathways are shared by 7/10 targets, 18 pathways are shared by 6/10 targets, and 40 pathways are shared by 5/10 targets (Fig. 7c). Comprehensive descriptions of all pathways affected by these targets are included in Supplementary Data 11. Moreover, we found an interesting association between *JMJD6* (as well as *NUDT2* and *NDRG4* among the 10 validated targets) and allele dosage. These 3 key driver genes are significantly associated with SNPs in their promoter regions (*cis*-eQTLs) in the MAYO and ROSMAP cohorts, further indicating that these genes may be actionable targets for AD therapeutic development.

## Discussion

AD is the most common neurodegenerative disease in the world, affecting millions of people worldwide. In the United States alone, an estimated 5.8 million Americans are currently living with AD dementia and this number is anticipated to reach 13.8 million by 2025^149^. Previous studies of LOAD pathogenesis using multi-omic data have identified numerous targets^21–24,68^. However, although neurons are the principal cell type affected by AD etiology, the molecular mechanisms and therapeutic targets for AD revealed by these studies are not specific to neurons due to a lack of large-scale scRNAseq data on neurons in AD. Thus, a comprehensive characterization of neuron-specific gene regulatory networks with association to AD is crucial to provide insight into the underlying causes of this disorder.

Here, we employed a self-developed computational systems biology approach to model AD neuronal gene regulatory networks, with which we identified upstream regulators (key drivers) in neurons that contribute to AD pathology. In our pipeline, we employed PSEA to deconvolute RNAseq data from brain region-specific tissue in the MAYO and ROSMAP cohorts into the five major cell types in the CNS including neurons, microglia, astrocytes, endothelial cells, and oligodendrocytes. In this study, we focused on the neuron-specific gene expression data and performed basic bioinformatics analyses including DE analysis, eQTL identification, co-expression module networks, and pathway enrichment analysis, followed by construction of causal network models and key driver gene identification.

From the network models, we identified a total of 1563 neuronal key drivers which may represent potential therapeutic targets. We used an unbiased ranking approach to prioritize 19 predicted key drivers for in vitro experimentation and tested the effects of their knockdown on the central components of the pathological hallmarks of AD, Aβ peptides (Aβ38, Aβ40, Aβ42) and phosphorylated tau protein, in a human iN system. We validated 10 targets which affected Aβ (*JMJD6*, *NSF*, *NUDT2*, *DCAF12*, *RBM4*, *YWHAZ*, *NDRG4*, and *STXBP1*) and/or tau/p-tau levels (*JMJD6*, *FIBP*, and *ATP1B1*). Only *YWHAZ* has been previously linked to AD through expression and mechanistic studies^150–154^, while others have not yet been studied. Our findings of alterations to the neurotoxic ratios of both Aβ42 to Aβ40 and p231-tau to tau suggest therapeutic potential to both early and later stages of disease considering known patterns of pathology development in AD^155^.

Most interestingly, we identified that knockdown of *JMJD6* (Jumonji Domain Containing 6, Arginine Demethylase and Lysine Hydroxylase) significantly increased both Aβ42 to 40 and p231-tau to tau ratios, suggesting therapeutic relevance to multiple stages of AD pathology. *JMJD6* belongs to the JmjC domain-containing family, catalyzes protein hydroxylation and histone demethylation, and appears to interact with distinct molecular pathways through epigenetic modifications of the genome^156,157^. JMJD6 is expressed in many tissues throughout the body, including the brain according to the Human Protein Atlas^158^, but very little is known about its role in the brain or in neurodegenerative disease. However, based on its known role in epigenetic regulation, it is expected that reduction of *JMJD6* expression may result in widespread changes in gene expression. Indeed, consistent with this prediction, we observed expression changes in a large number of genes following neuronal knockdown of *JMJD6*, including alteration of the expression of 3 other key driver targets of interest highlighted in this study (*NDRG4*, *ATP6V1A*, and *NSF*).

We recognize that one caveat of our experimental system is that neurons in a dish are not the same as neurons present in the aged AD brain; however, neurons in vitro do represent a powerful system for interrogating molecular connections between gene expression and proteins relevant to AD (namely, Aβ and tau). We recently showed that neurons derived from >50 different individuals show concordance between their levels of specific Aβ peptides and p-tau species and levels of these same proteins expressed in the brains of the same individuals^72^. Further, we showed concordance between protein and RNA module expression between the iPSC-derived neurons and the brain tissue of the same people. Taken together, these results suggest that in spite of the reductionist nature of the system and the lack of aging, molecular networks are captured within the cells in vitro that are reflected in changes in Aβ and tau. Here, we employ this same experimental system to show that targeted reduction of *JMJD6* levels in human neurons induces effects on Aβ ratios and tau levels and phosphorylation.

Through our network models, we also discovered REST and VGF as two shared downstream effectors of the 10 validated targets, which may potentially explain the observed modulation of AD pathology. REST is a known master regulator of neurogenesis via epigenetic mechanisms, apoptosis, and oxidative stress^147,148^ whose loss has been causally linked to Alzheimer’s disease^159,160^. Additionally, recent studies have identified an association between changes in the epigenome, such as DNA methylation and histone modification, with changes in cognitive functions such as learning and memory^161–170^. Thus, dysregulation of epigenetic mechanisms through modulation of the targets may play a role in the pathogenesis of AD^162,171^. VGF is also a target of interest which was recently found to partially rescue memory impairment and neuropathology in 5xFAD mice^68^. Overexpression of VGF increased activated BDNF receptor levels as well as adult hippocampal neurogenesis, which in turn regulated postsynaptic protein PSD-95 and improved cognition in the 5xFAD mice^68^. Our pathway enrichment analysis confirmed that all 10 key drivers and their downstream genes in the network models were also significantly enriched for a variety of convergent and unique downstream cellular processes and functions which may explain additional molecular mechanisms at play, including vesicle-mediated membrane trafficking (common downstream of 8 targets); axon guidance, intra-Golgi trafficking, and retrograde Golgi-to-ER trafficking (common of 7 targets); and signaling pathways for sphingolipids, prolactin, BDNF/NTRKs, EGF-EGFR, TNFα, RHO GTPases, TP53, receptor tyrosine kinases (RTKs), and ER-to-Golgi transport (common of 6 targets).

In summary, our innovative computational systems biology approach using predictive network modeling has identified 10 targets which significantly modulate AD pathology via regulation of a variety of downstream pathways. These processes involve a wide spectrum of cellular pathways and possible mechanisms, and our results offer insights into potential therapeutic targets for drug discovery in AD.

## Methods

### Obtaining RNAseq and genome-wide genotype datasets

MAYO temporal cortex RNAseq data (id: syn3163039) and genome-wide genotype data (id: syn8650953) were downloaded from the AMP-AD knowledge portal hosted on Synapse.org (doi:10.7303/syn2580853). The ROSMAP dorsolateral prefrontal cortex RNAseq data (id: syn4164376), genotypes (id: syn3157325), and clinical covariates (id: syn3191087) were downloaded from Synapse.org using the synapseClient R library^172^.

### Study participants and ethical statements

The MAYO dataset includes 278 subjects: 84 with AD, 84 with progressive supranuclear palsy (PSP), 80 cognitively normal controls, and 30 with pathologic aging^83–85,173^ (see Supplementary Note 1 for more information on diagnostic criteria relevant to this study). All AD and PSP subjects along with 65 control subjects were from the Mayo Clinic Brain Bank; all pathologic aging and remaining control subjects were from the Banner Sun Health Institute. The Mayo RNAseq Study was approved by the Mayo Clinic Institutional Review Board. All human subjects or their next of kin provided informed consent. All subjects were North American Caucasians. In this study, we analyzed a total of 266 MAYO subjects with matched RNAseq and genome-wide genotype data, including 79 AD subjects and 76 cognitively normal subjects. All disease subjects had ages at death ≥60 years; a more relaxed age cutoff of ≥50 years was applied for control subjects to achieve a sample size similar to that of the AD subjects, but we note there were only two additional control subjects with age at death below 60. We performed rigorous statistical testing to demonstrate that these samples are well balanced with respect to age at death (*p*-value = 0.57) as well as sex (*p*-value = 0.24) (Supplementary Fig. 7a, c).

The ROSMAP dataset includes the Religious Orders Study (ROS) and the Memory and Aging Project (MAP)^174^, which are both longitudinal clinical-pathologic cohort studies of aging and dementia run by the Rush Alzheimer’s Disease Center in Chicago, IL. All participants enroll without known dementia and agree with informed consent to annual clinical evaluation and brain donation. Each sample is associated with a cognitive diagnosis of: not cognitively impaired, mild cognitive impairment, or AD (see Supplementary Note 2 for more information on diagnostic criteria). The ROS and MAP studies were each approved by an Institutional Review Board of Rush University Medical Center. In this study, we analyzed a total of 612 ROSMAP subjects with matched RNAseq and genome-wide genotype data, including 212 AD subjects and 194 cognitively normal subjects.

### MAYO RNAseq, data processing, and quality control

RNA extraction, library preparation, and sequencing of the temporal cortex samples were conducted at the Mayo Clinic Medical Genome Facility Genome Analysis Core, as previously described^175^ (see also Supplementary Note 3 for more information). Only samples with an RNA integrity number ≥5.0 were included in this study. Briefly, all samples underwent 101 base-pair, paired-end sequencing on Illumina HiSeq2000 instruments. Base-calling was performed using Illumina’s Real-Time Analysis 1.17.21.3. FASTQ sequence reads were aligned to the human reference genome using TopHat 2.0.12^176^ and Bowtie 1.1.0^177^, and Subread 1.4.4 was used for gene counting^178^. FastQC^179^ was used for quality control (QC) of raw sequence reads, and RSeQC^180^ was used for QC of mapped reads.

All MAYO RNAseq samples had percentage of mapped reads ≥85%. Raw read counts were transformed to counts per million (CPM), log2 normalized, and normalized using Conditional Quantile Normalization (CQN) via the Bioconductor package^181^, accounting for sequencing depth (calculated as the sum of reads mapped to genes), gene length, and GC content (calculated via Repitools^182^ in the Bioconductor package). Genes with non-zero counts across all samples were retained and principal component analysis was performed using the prcomp function in R. Principal components 1 and 2 were plotted and no outliers (>6 SD from mean) were identified.

### ROSMAP RNAseq, data processing, and quality control

BAM files^174^ were sorted using samtools^183^ and converted to FASTQ files using the SamToFastq function^184^. RAPiD^185^ was used to generate a count matrix for the gene expression data and a vcf file for each sample aligned to hg19 from the FASTQ files. Read count expression data was normalized using log2 counts per million (CPM) and the TMM method^186^ was implemented in edgeR^187^. Genes with over 1 CPM in at least 30% of the experiments were retained. We then used precision weights as implemented in the voom function from the limma^188^ R package to further normalize the gene counts.

Regarding the ROSMAP cohort, it has been noted that the range of age at death is broad but restricted to the older segment of the age distribution of the North American population and that age and sex are important confounders when performing any analyses of ROS and MAP data^88^. We observed this variance in the age at death (*p*-value < 0.05) but found no significant difference in sex among the ROSMAP subjects used in our analysis (*p*-value = 0.072) (Supplementary Fig. 7b, d). To address the imbalanced age distribution, we later performed covariate adjustment for age (together with other covariates, see section ‘Deconvolution of RNAseq data into neuron-specific expression residuals’ below), and we confirmed removal of the effects of age and other confounding variables by variance partition analysis before and after covariate adjustment (Supplementary Fig. 2b, d).

### Genome-wide genotype data and quality control

Whole-genome genotyping of MAYO subjects was performed at the Mayo Clinic Medical Genome Facility Genome Analysis Core using the Illumina Infinium HumanOmni2.5-8 Kit (see also Supplementary Note 4 for more information). Whole genome genotype calls were made using the auto-calling algorithm in Illumina’s BeadStudio 2.0 software, after which they were converted into PLINK formats for analysis^189^. Samples were removed if they had discordant sex, heterozygosity rates >3 SD from the mean, or apparent relation. The dataset was filtered to include only autosomal SNPs and to remove complex genomic regions regions (chr8:1-12,700,000; chr2:129,900,001-136,800,000; chr17:40,900,001-44,900,000; and chr6:32,100,001-33,500,000). Linkage disequilibrium was pruned using the SNPRelate (v1.4.2) package in R^190^, implementing a linkage disequilibrium threshold of 0.15 and a sliding window of 1E-07 base pairs. Remaining SNPs and subjects were analyzed using EIGENSOFT^191^ for population outliers. See Supplementary Note 5 for more details regarding MAYO sample exclusion.

ROSMAP subject genotype data was processed using PLINK 2.0^192^. Positions were converted from hg18 to hg19 (http://genome.ucsc.edu/cgi-bin/hgLiftOver) and resulting genotype files were sorted using Picard^184^. Samples were removed using PLINK 2.0 if they had variants with >2% missing values, minor allele frequency <1%, Hardy-Weinberg equilibrium <10E-6, or inbreeding coefficient >0.15.

### Genotype data imputation

1000 Genomes Project^193^ data and IMPUTEv2^194^ were used to impute untyped variants. Imputed variants were removed if they failed any of the previously listed quality control criteria or had information scores <0.6. After imputation, we had 7,132,687 variants in MAYO and 9,333,139 variants in ROSMAP.

### Deconvolution of RNAseq data into neuron-specific expression residuals

Residuals were obtained for each RNAseq dataset by adjusting for covariates using the limma R package^188^. For MAYO, expression residuals were obtained by correcting for the effects of technical confounding factors (i.e., sequencing batch), sample-specific variables (RNA integrity number, exonic mapping rate, source of tissue), and patient-specific covariates (sex, age at death, *APOE* genotype). For ROSMAP, we adjusted for a slightly different set of covariates due to a greater number of recorded measurements available: study (ROS or MAP), sequencing batch, post-mortem interval, RNA integrity number, exonic mapping rate, sex, educational attainment, *APOE* genotype, and age at death. For both MAYO and ROSMAP data, we computed the exonic mapping rate using RNAseQC^195^.

We additionally adjusted for previously published single-gene biomarkers derived at the protein level under AD for the five major cell types in the CNS: neurons, *ENO2*^96^; microglia, *CD68*^97^; endothelial cells, *CD34*^99^; astrocytes, *GFAP*^98^; and oligodendrocytes, *OLIG2*^100–102^. To obtain expression residuals that mimic expression patterns seen in neurons, for every gene, we added the *ENO2* effects estimated by the linear regression models back to the expression residuals. Comparing the variance of normalized gene expression before and after covariate adjustment, we confirmed removal of the effects from confounding variables (Supplementary Fig. 2), allowing us to conclude that the residual results are unbiased and robust against these adjusted covariates.

The final neuron-specific expression residual data available for further analysis included 19,885 genes from 155 individuals in MAYO (79 AD, 76 cognitively normal) and 20,276 genes from 406 individuals in ROSMAP (212 AD, 194 cognitively normal), with 18,408 genes common to both datasets (Fig. 1b, Fisher’s exact test, *p*-value = 0); this is comparable to processed residuals of the same cohorts on the AMP-AD knowledge portal (https://adknowledgeportal.synapse.org).

### Rationalization and validation of single-gene biomarkers for bulk-tissue RNAseq deconvolution

Our rationale for using single-gene biomarkers over multi-gene biomarkers derived from scRNAseq data was manifold. First, multi-gene biomarkers derived from various scRNAseq studies in control human brains^54–58^ (Supplementary Data 12) show no significant overlap among themselves, indicating a lack of robustness and consensus in these biomarkers derived from scRNAseq studies (Supplementary Fig. 3a). Second, PCA analysis shows a prominent overlap of scRNAseq biomarker expression across different cell types in MAYO and ROSMAP AD data, indicating that the majority of scRNAseq-derived biomarker gene expression is convoluted and reflecting potential interactions between different cell types under the AD condition (Supplementary Fig. 3b, c; Supplementary Data 12). Furthermore, there is significant overlap between scRNAseq-derived biomarkers and AD therapeutic targets in the AMP-AD Agora list^142^ (Supplementary Fig. 3j, k). This overlap is more significant than randomly selected genes from the background overlapping with the Agora list, indicating that scRNAseq-derived biomarkers may play a role in AD pathology. For these reasons, all or a random subset of scRNAseq-derived cell type biomarkers are not ideal for adjusting the bulk-tissue gene expression variance by PSEA.

By contrast, our single-gene biomarkers are derived directly at the protein level under AD conditions and have been validated by other groups^83,96–102^. They show no overlap with AD therapeutic targets in the Agora list, thus making them good candidates for PSEA. Furthermore, our neuron-specific residual derived from the single-gene biomarker *ENO2* is significantly correlated with pseudo neuron-specific residuals derived from a randomly selected subset of scRNAseq biomarkers (Supplementary Fig. 3h, i; Supplementary Note 6), indicating that our neuron-specific residual represents a robust neuronal component in the bulk-tissue RNAseq data for neuron-specific therapeutic target discovery in LOAD.

### Computational analyses of neuron-specific gene expression data

eQTL analysis was performed using the R package MatrixEQTL v2.1.1^196^ using quality-controlled genotypes and normalized and covariate-adjusted cell type-specific expression residuals. *cis*-eQTL analysis considered markers within 1 Mb of the transcription start site of each gene. False discovery rates (FDR) were computed using the Benjamini–Hochberg procedure^197^.

DE analysis to interrogate the cell type-specific residual expression data for genes differentially expressed between AD cases and healthy controls was performed using linear models implemented in the limma R package^188^. Significance was assessed using FDR < 0.05. We note that the log-fold change thresholds in Fig. 2a, b are for visualization only and were not used in the analysis in any way.

For pathway enrichment analyses, we downloaded pathways from ConsensusPathDataBase^105^. For each given set of genes, we performed enrichment analysis of each pathway over the set by Fisher’s exact test with *p* < 0.05.

Co-expression networks were constructed using the coexpp R package^198^. A soft thresholding parameter value of 6.5 was used to power the expression correlations. Seeding gene lists for the predictive networks were obtained by selecting genes in co-expression modules that were statistically enriched (FDR < 0.05) for DE genes or neuronal cell markers^57^.

To perform key driver analysis, we used the KDA R package^82^ (version 0.1, available at http://research.mssm.edu/multiscalenetwork/Resources.html). The package first defines a background sub-network by looking for a neighborhood k-step away from each node in the target gene list in the network. Then, stemming from each node in this sub-network, it assesses the enrichment in its k-step (k varies from 1 to K) downstream neighborhood for the target gene list. In this analysis, we used *K* = 6. Prioritization of key drivers for subsequent assessment was determined by ranking their impact score and robustness score (described in Supplementary Notes 7 and 8, respectively).

Predictive network modeling was performed according to detailed methods described in our recent publications^23,59,60,199,200^ as well as methodology patent US11068799B2.

For pathway analysis, we used the PathFinder method^60^ which is based on the classical Depth-First Search algorithm^201^. The goal of PathFinder is to expand the initial target gene set by including genes in the background network located in the paths connecting input genes. Since the background network could contain directed and undirected edges, we transformed the undirected edges into two edges with the same two end nodes but different directions. We did not allow these two edges to form a loop and simultaneously appear in one path. The Depth-First Search algorithm starts from one input gene and stops if the length of path it explores reaches K or if the path arrives at a node without a valid child node. Whenever any of the stop criteria above was satisfied, we checked whether the path contained at least two input genes. If not, the path was discarded. Otherwise, among all the input genes in the path, we determined the target gene with the maximum distance to the starting input gene, and all the nodes between this gene and the starting input gene were then included in the seeding gene list for the network. In practice, we ran Depth-First Search for each input gene and combined the results to obtain the final network seeding gene list.

### iPSC maintenance and induced neuron differentiation

The human control iPSC line YZ1 was obtained from the University of Connecticut Stem Cell Core facility and was maintained in StemFlex Medium (Thermo Fisher Scientific, Waltham, MA). Induced neurons (iNs) were generated as described^71,72,143^, with minor modifications. Briefly, iPSCs were plated in mTeSR1 media (STEMCELL Technologies, Vancouver, Canada) at a density of 95 K cells/cm^2^ on Matrigel-coated plates (Corning Inc., Corning, NY) for viral transduction. Media was changed from StemFlex to mTeSR1 as we found better transduction viability with mTeSR1. Viral plasmids were obtained from Addgene (plasmids #19780, 52047, 30130; Watertown, MA). FUdeltaGW-rtTA was a gift from Konrad Hochedlinger (Addgene plasmid #19780), and Tet-O-FUW-EGFP (Addgene plasmid #30130) and pTet-O-Ngn2-puro (Addgene plasmid #52047) were gifts from Marius Wernig. Lentiviruses were obtained from ALSTEM (Richmond, CA) with ultra-high titers and used at the following concentrations: pTet-O-NGN2-puro: 0.1 μl/50 K cells; Tet-O-FUW-eGFP: 0.05 μl/50 K cells; Fudelta GW-rtTA: 0.11 μl/50 K cells. Transduced cells were dissociated with Accutase (Gibco, Thermo Fisher Scientific) and plated onto Matrigel-coated plates at 50 K cells/cm^2^ in mTeSR1 (day 0). On day 1, media was changed to KSR media with doxycycline (2 μg/ml, Sigma-Aldrich, St. Louis, MO). Doxycycline was maintained in the media for the remainder of the differentiation. On day 2, media was changed to 1:1 KSR:N2B media with puromycin (5 μg/ml, Gibco). On day 3, media was changed to N2B media with 1:100 B27 supplement and puromycin (10 μg/ml). Puromycin was maintained at this concentration in the media for the remainder of the differentiation. From day 4 onwards, cells were cultured in NBM media with 1:50 B27 and BDNF, GDNF, CNTF (10 ng/ml each, PeproTech, Rocky Hill, NJ), plus doxycycline and puromycin as described. iNs were not co-cultured with human or primary rodent astrocytes in this study. See Supplementary Note 9 for media formulations.

### Lentiviral transduction of induced neurons

At day 17 of differentiation, neurons were transduced with lentiviruses encoding shRNA constructs against selected targets (Broad Institute, Cambridge, MA), as described in ref. ^22^. For each round of experiments, two controls were included: a lentivirus expressing the pLKO vector without an shRNA (empty) or else not transduced (fresh media only). iNs were transduced with a 1:1 ratio of media to lentivirus. Following ~18 h of incubation, media containing virus was removed and replaced with fresh media, and cells were incubated for an additional 96 h. On day 22 of differentiation, conditioned media was then collected and stored at −20 °C for Aβ analyses, and cells were lysed either for RNA purification or protein harvest. Gene knockdowns were confirmed by qPCR.

### Aβ ELISA

Aβ present in the conditioned media was measured by the 6E10 Aβ Peptide Panel Multiplex ELISA (Meso Scale Discovery, Rockville, MD) following manufacturer instructions. Briefly, conditioned media from transduced cells were incubated in pre-blocked wells along with detection antibody solution. Plates were read using an MSD SECTOR Imager 2400 and resulting peptide concentrations were normalized to total protein in the cell lysate per well measured using the Pierce BCA Protein Assay Kit (Thermo Fisher Scientific). Data for each shRNA knockdown were additionally normalized to the average of control conditions for each parameter measured. To then compare each target shRNA to the control condition for each parameter, Dunnett’s T3 tests for multiple comparisons were performed in Prism 9.0.

### Tau ELISA

Protein was extracted from iNs by lysis in NP-40 lysis buffer (1% NP40, 0.5 M EDTA, 5 M NaCl, 1 M Tris) containing cOmplete protease inhibitors and phosSTOP (Roche, Penzberg, Germany). Lysates were analyzed using the Multi-Spot Phospho (Thr 231)/Total Tau ELISA (Meso Scale Discovery) following manufacturer instructions. Briefly, lysates were incubated in pre-blocked wells for 1 hr prior to detection antibody application for 1 hr. Plates were read using an MSD SECTOR Imager 2400 and resulting concentrations were again normalized to total protein in the cell lysate per well (Pierce BCA Protein Assay Kit) and data for each shRNA knockdown were normalized to the average of control concentrations for each parameter. To then compare each target shRNA to the control condition for each parameter, Dunnett’s T3 tests for multiple comparisons were performed in Prism 9.0.

### RNA sequencing of induced neurons

For iNs, at least 250 ng of total RNA input was oligo(dT) purified using the PureLink RNA Mini Kit (Invitrogen), then double-stranded cDNA was synthesized using SuperScript III Reverse Transcriptase (Invitrogen) with random hexamers. RNA integrity number >9 was confirmed using the Agilent 4200 TapeStation system (Agilent Technologies). RNAseq on the shRNA-treated iNs was performed by Functional Genomics Core at the University of Arizona at a depth of 30 million single-end reads (100 bp long). The RNAseq data (GSE228156) was QCed and processed with the same steps as outlined in the Methods section “ROSMAP RNAseq, data processing, and quality control”.

### Statistics and reproducibility

All statistical analyses were performed in R Foundation for Statistical Computing, version 3.2.3, unless otherwise noted. Genotyping and RNAseq data was pre-processed and normalized (range of *N* = 266 for MAYO and *N* = 612 for ROSMAP). The normalized gene expression counts were then corrected for covariates as described in ‘Methods’. The residual values were then used to perform DE, co-expression module, and network analysis.

### Reporting summary

Further information on research design is available in the Nature Portfolio Reporting Summary linked to this article.

## Supplementary information

Supplementary Information

Description of Additional Supplementary Files

Supplementary Data 1

Supplementary Data 2

Supplementary Data 3

Supplementary Data 4

Supplementary Data 5

Supplementary Data 6

Supplementary Data 7

Supplementary Data 8

Supplementary Data 9

Supplementary Data 10

Supplementary Data 11

Supplementary Data 12

Supplementary Data 13

Supplementary Data 14

Supplementary Data 15

Supplementary Data 16

Reporting summary

## Data Availability

The datasets analyzed in this study are available on the AMP-AD knowledge portal hosted on Synapse.org (doi:10.7303/syn2580853) with the following accessions: MAYO temporal cortex RNAseq data (syn3163039), MAYO genome-wide genotype data (syn8650953), ROSMAP DLPFC RNAseq data (syn4164376), ROSMAP genotypes (syn3157325), and ROSMAP clinical covariates (syn3191087). Requests for ROSMAP data can also be made at www.radc.rush.edu/. The RNAseq data generated from target knockdown experiments can be downloaded from GEO (GSE228156). Source data underlying Figs. 2–5 are presented in Supplementary Data 13–16, respectively.

## Code availability

The code for network construction can be obtained at labs.icahn.mssm.edu/zhulab/software/. The software Co-expp for co-expression module construction can be obtained from bitbucket at https://bitbucket.org/multiscale/coexpp/src/master/. The network software can be obtained from https://labs.icahn.mssm.edu/zhulab/?s=rimbanet.
