# Supplementary material for: Predictive network analysis identifies JMJD6 and other potential key drivers in Alzheimer’s disease
Source: Commun Biol. 2023 May 15;6:503. doi: 10.1038/s42003-023-04791-5 (PMC10185548; doi:10.1038/s42003-023-04791-5)
Supplement: Supplementary file 1 — Supplementary Information [file 42003_2023_4791_MOESM1_ESM.pdf]

# Supplementary Information

## Predictive network analysis identifies *JMJD6* and other potential key drivers of Alzheimer's disease

Julie P. Merchant[1,a]*, Kuixi Zhu[2]*, Marc Y.R. Henrion[3,4], Syed S.A. Zaidi[2], Branden Lau[2,5], Sara Moein[2], Melissa L. Alamprese[2], Richard V. Pearse II[1], David A. Bennett[6], Nilüfer Ertekin-Taner[7,8], Tracy L. Young-Pearse[1,9 #], Rui Chang[2,10,11,12 #]

[1]Ann Romney Center for Neurologic Diseases, Brigham and Women's Hospital and Harvard Medical School, Boston, MA, USA.
[2]The Center for Innovation in Brain Sciences, University of Arizona, Tucson, AZ, USA.
[3]Liverpool School of Tropical Medicine, Pembroke Place, Liverpool, Pembroke Place, L3 5QA, UK.
[4]Malawi - Liverpool - Wellcome Trust Clinical Research Programme, PO Box 30096, Blantyre, Malawi.
[5]Arizona Research Labs, Genetics Core, University of Arizona, Tucson, AZ, USA.
[6]Rush Alzheimer's Disease Center, Rush University Medical Center, Chicago, IL, USA.
[7]Department of Neuroscience, Mayo Clinic Florida, Jacksonville, FL, USA.
[8]Department of Neurology, Mayo Clinic Florida, Jacksonville, FL, USA.
[9]Harvard Stem Cell Institute, Harvard University, Boston, MA, USA.
[10]Department of Neurology, University of Arizona, Tucson, AZ, USA.
[11]INTelico Therapeutics LLC, Tucson, AZ, USA.
[12]PATH Biotech LLC, Tucson, AZ, USA.
[a]Present address: Neuroscience Graduate Group, University of Pennsylvania Perelman School of Medicine, Philadelphia, PA, USA.

*These authors contributed equally: Julie P. Merchant, Kuixi Zhu.

#These authors jointly supervised this work: Tracy L. Young-Pearse, Rui Chang.

Corresponding authors: Tracy L. Young-Pearse (tpearse@bwh.harvard.edu), Rui Chang (ruichang@email.arizona.edu).

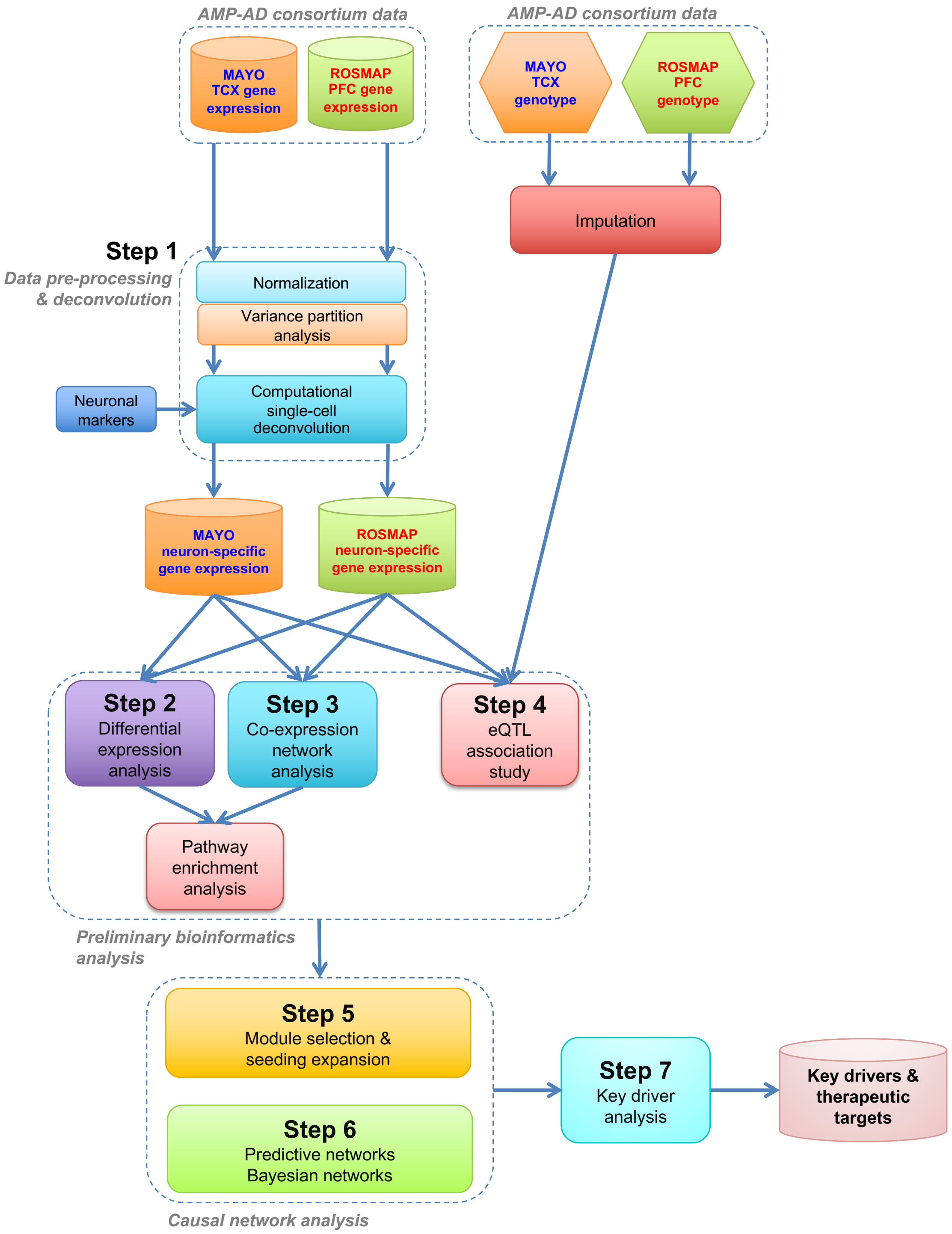


**Supplementary Figure 1. Workflow of our network analysis pipeline**. We integrated two independent brain transcriptome and genome-wide genotype datasets (MAYO and ROSMAP) to construct neuron-specific predictive networks of AD and predict key drivers (potential therapeutic targets) associated with AD pathology. TCX: temporal cortex; PFC: prefrontal cortex; eQTL, expression quantitative trait locus.

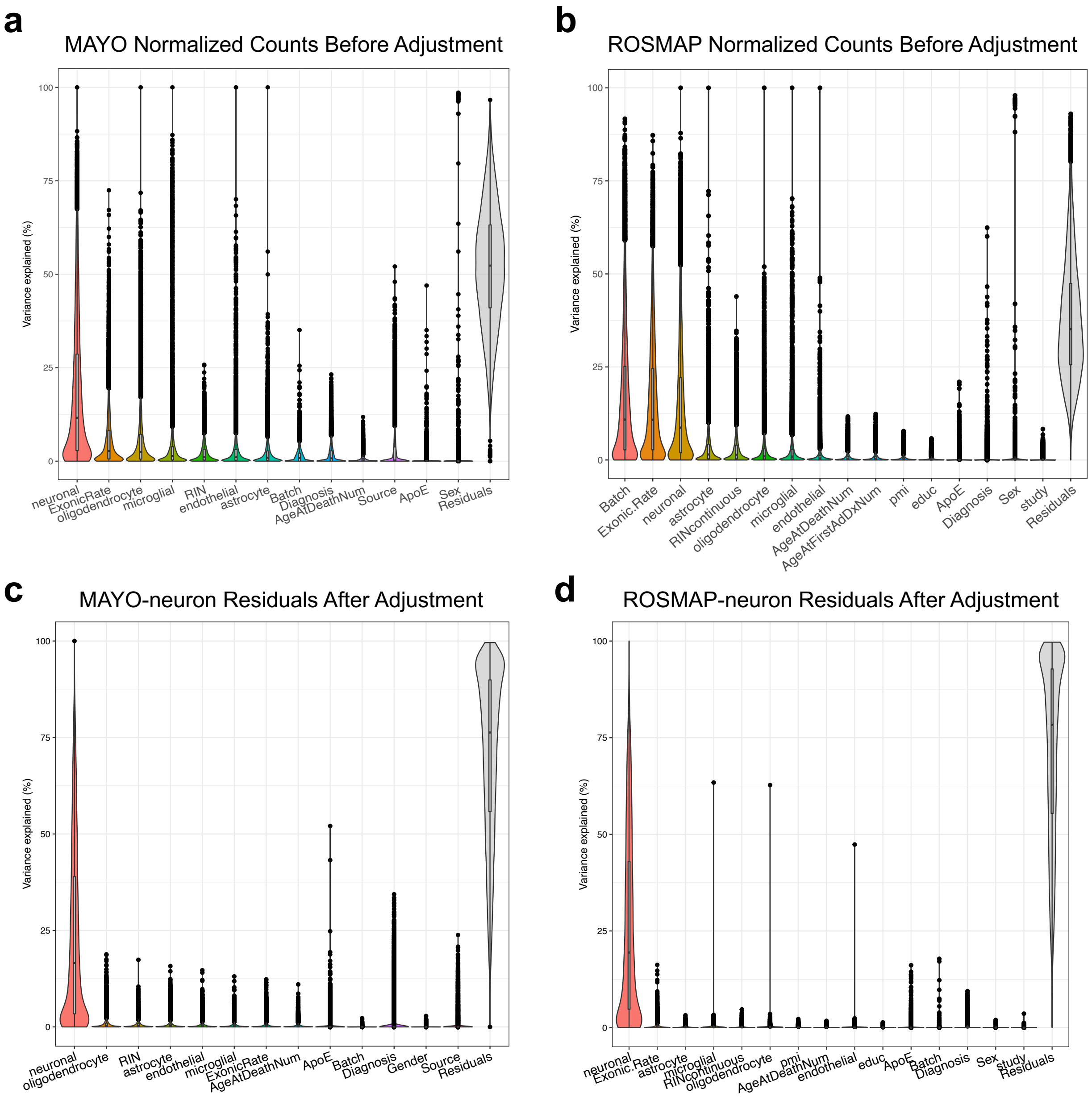


**Supplementary Figure 2. Variance partition analysis of MAYO and ROSMAP gene expression data**. (**a,b**) Gene expression variance partition analysis (VPA) of bulk-tissue RNAseq data in the MAYO (**a**) and ROSMAP (**b**) cohorts _before_ deconvolution and covariate adjustment reveals a prominent effect of cell type on bulk-tissue gene expression in the brain. *ENO2*, *CD68*, *GFAP*, *CD34*, and *OLIG2* were used as cell type-specific marker genes for neurons, microglia, astrocytes, endothelial cells, and oligodendrocytes, respectively. (**c,d**) VPA on neuron-specific gene expression residuals in the MAYO (**c**) and ROSMAP (**d**) cohorts _after_ deconvolution and covariate adjustment demonstrates that the neuron-specific residuals capture the neuronal component (variance) and that the effects of other covariates and other cell types in the brain are removed. Violin Plots in (a-d) are divided into four quartiles. The middle quartiles are represented by a box that contains 50% of the data and the median value. The upper and lower quartiles contain the maximum and minimum values and the remaining 50% of the data.

ExonicRate: exonic mapping rate; RIN or RINcontinuous: RNA integrity number; AgeAtFirstADDxNum: age at first AD diagnosis; pmi: post-mortem interval; educ: education.

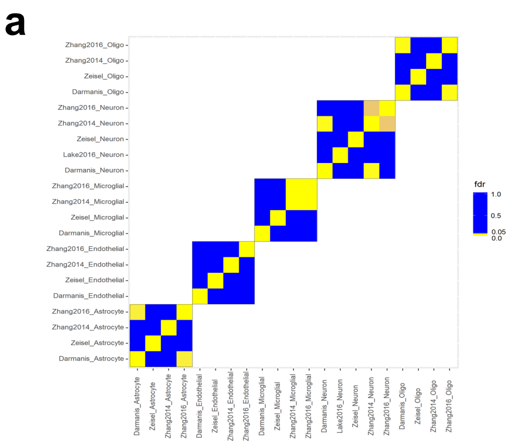


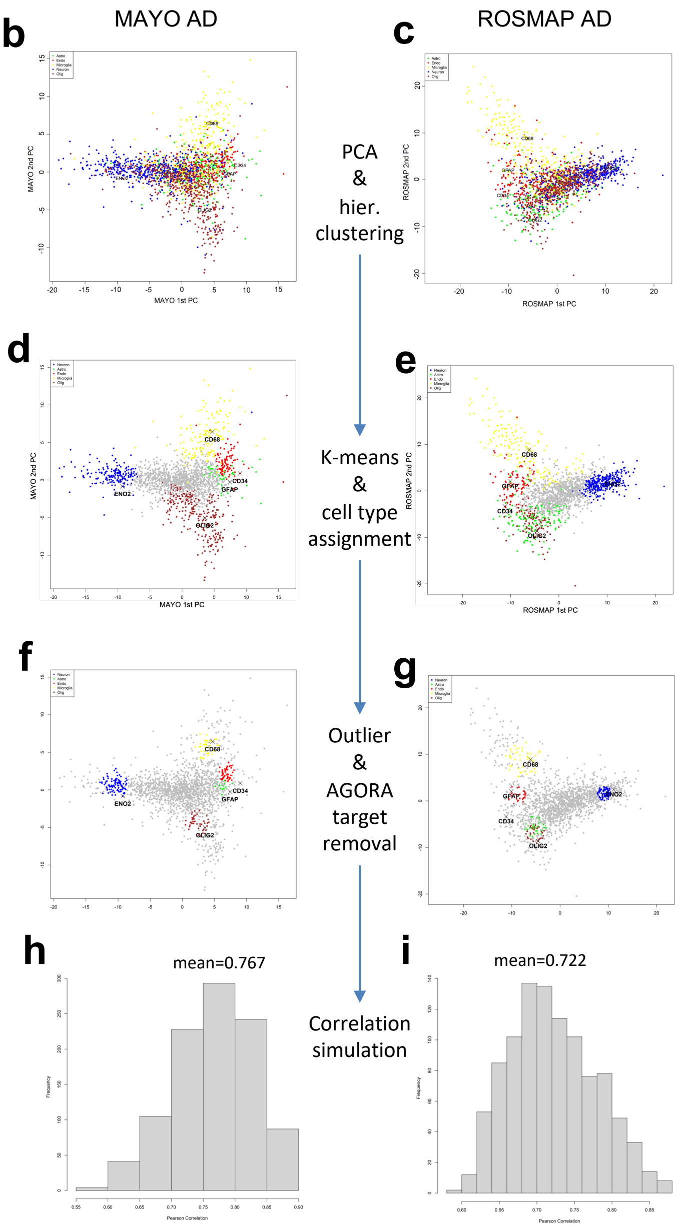


**j**

| Cell Type | Overlap with AMP-AD Agora | P-value |
|---|---|---|
| Astrocyte | 6.57% (19) | 2.18E-03 |
| Endothelial | 3.36% (12) | 0.447 |
| Microglial | 8.61% (34) | 1.19E-06 |
| Neuron | 7.23% (41) | 4.95E-07 |
| Oligodendrocyte | 7.29% (38) | 3.43E-06 |

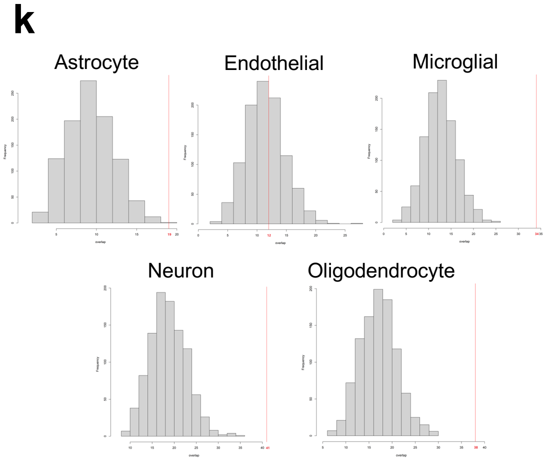


**Supplementary Figure 3. Robustness analyses of our neuron-specific residuals derived from single biomarkers by population-specific expression analysis (PSEA), for both the MAYO and ROSMAP datasets, compared to mulit-gene biomarkers derived from scRNAseq studies**. (**a**) Enrichment analysis of multiple multi-gene biomarker lists for the five main CNS cell types derived from various scRNAseq studies in control human brains – as referenced in [1-5] – shows no significant overlap among the studies. For each cell type, we compared the biomarker genes by pairing each scRNAseq study and calculating the significance of the overlap by Fisher's exact test with FDR<0.05, finding 1/0/1/1 significant pairs out of 6 study-pairs in astrocytic/endothelial/microglial/oligodendrocytic types and 2 significant pairs out of 10 study-pairs in neuronal types. (**b-i**) After creating a merged biomarker list for each cell type from the above scRNAseq studies (Supplementary Data 12), we then extracted

the gene expression matrix of the merged biomarkers from the MAYO and ROSMAP RNAseq datasets. Principal component analysis (PCA) shows a prominent overlap of scRNAseq biomarker expression across the five main CNS cell types in AD residuals from the MAYO (**b**) and ROSMAP (**c**) datasets after covariate adjustment. Hierarchical clustering was then performed to group all scRNAseq biomarkers in each PCA space into small bins, followed by K-means analysis to further group these bins into larger clusters and cell type assignment of each cell type to a distinct cluster for MAYO (**d**) and ROSMAP (**e**) along with an overlapping cluster (junk cluster) per dataset. Final clean clusters were obtained by removing outliers and Agora targets using cutoff=1xSD in MAYO (**f**) and ROSMAP (**g**). Finally, correlation simulation was performed on the final clusters in MAYO (**h**) and ROSMAP (**i**) to calculate the distribution of Pearson correlations between the residuals derived from our single biomarker approach and randomly subsampled biomarkers. See Supplementary Note 6 for more details on the analysis steps behind panels **b-i**. (**j**) Enrichment analysis via Fisher's exact test reveals significant (p-value<0.05) overlap between scRNAseq biomarkers and AMP-AD Agora targets for neurons, microglia, astrocytes, and oligodendrocytes. The value in parentheses represents the number of genes overlapping between each biomarker list and the AMP-AD Agora targets. (**k**) Compared to randomly selected genes from the background overlapping with the AMP-AD Agora list, the number of overlapping genes between scRNAseq biomarkers and AMP-AD Agora targets (red vertical line along the distribution) is significantly higher for four cell types, including neurons. For each cell type, we simulated a background distribution of overlap by randomly selecting the same number (as on the merged biomarker list; Supplementary Data 12) of background genes – taking the non-duplicate union of genes in MAYO and ROSMAP RNAseq data – and comparing the randomly generated pseudo biomarker list ot the AMP-AD Agora targets to generate an overlapping percentage. Significance was assessed by Fisher's exact test with FDR<0.05 and the p-value was calculated per cell type by comparing the true percentage to the background distribution.

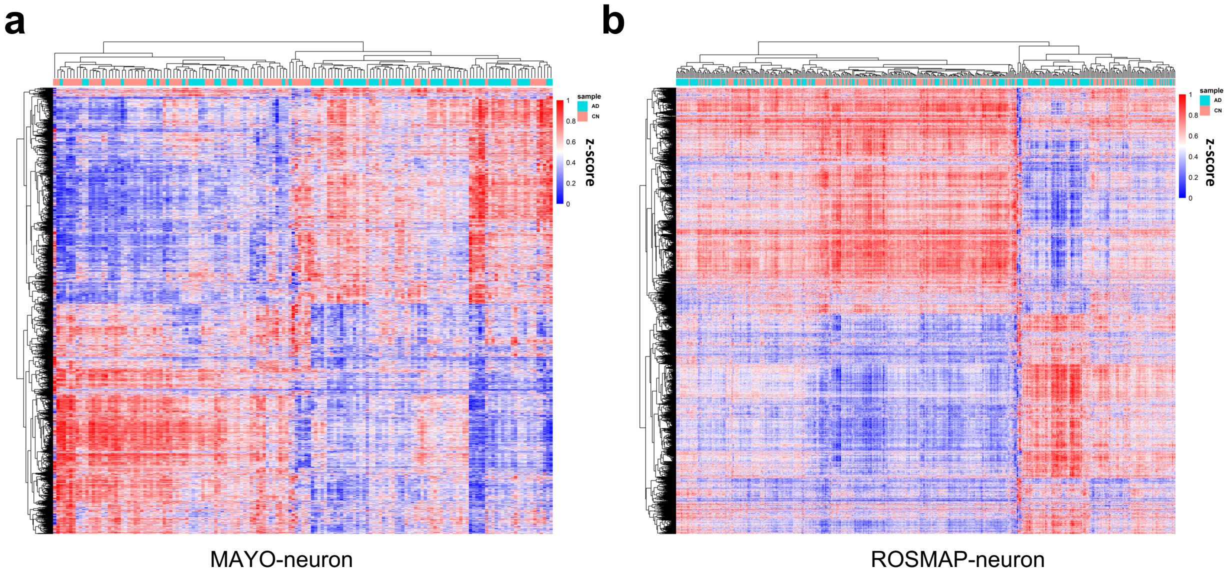


**Supplementary Figure 4**. **Neuron-specific differential expression signatures in AD**. (**a**,**b**) Heatmaps showing clusters of upregulated (red) and downregulated (purple) genes in the neuron-specific residuals of AD patients compared to cognitively normal (CN) controls in MAYO (**a**) and ROSMAP (**b**).

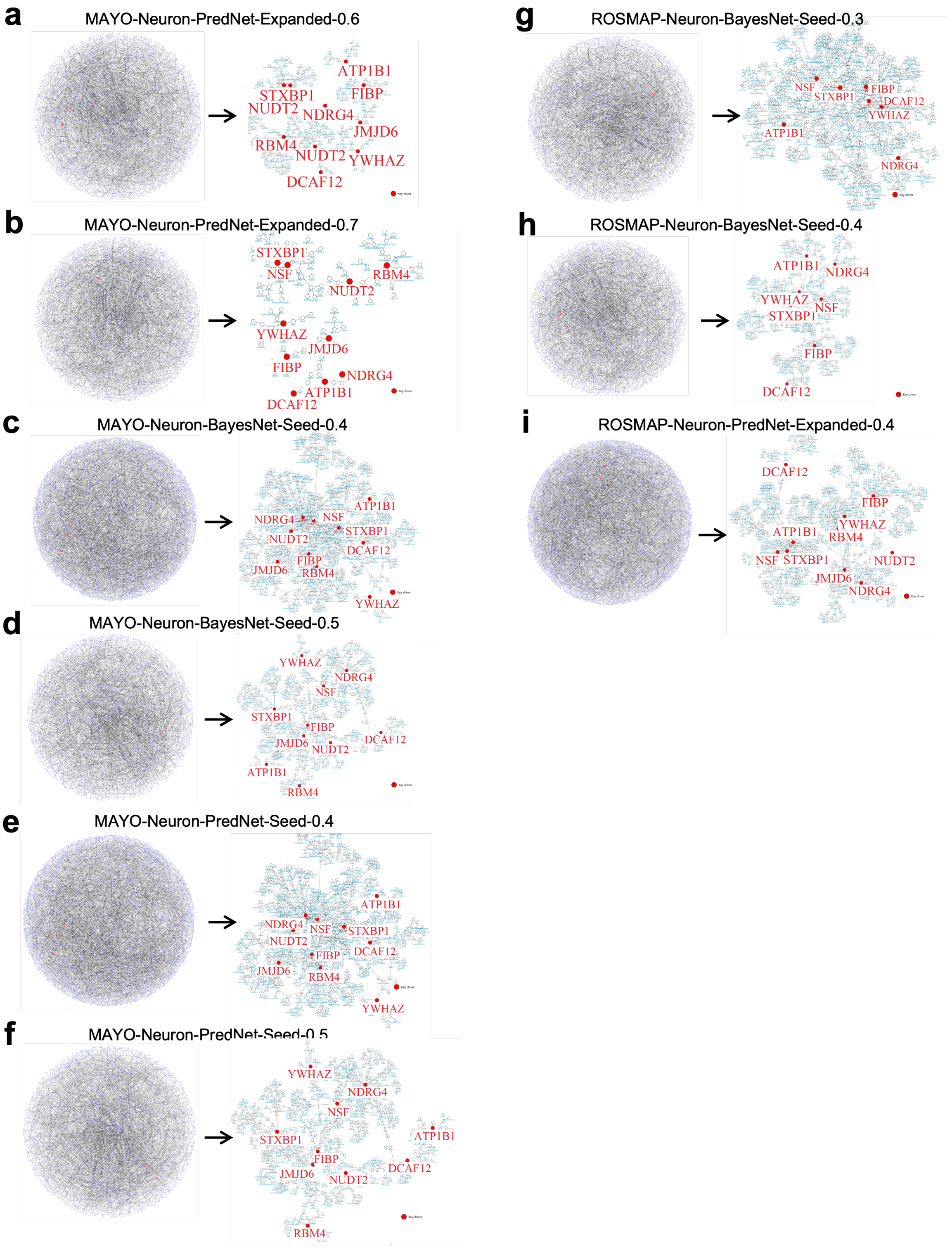


**Supplementary Figure 5. Remaining 9 of 11 neuron-specific network models**. Bayesian and predictive network models derived from the MAYO and ROSMAP seeding and expanded gene sets, with the downstream subnetworks of the 10 validated targets highlighted. Posterior probability cut-offs used to build each network model are indicated in each panel's title.

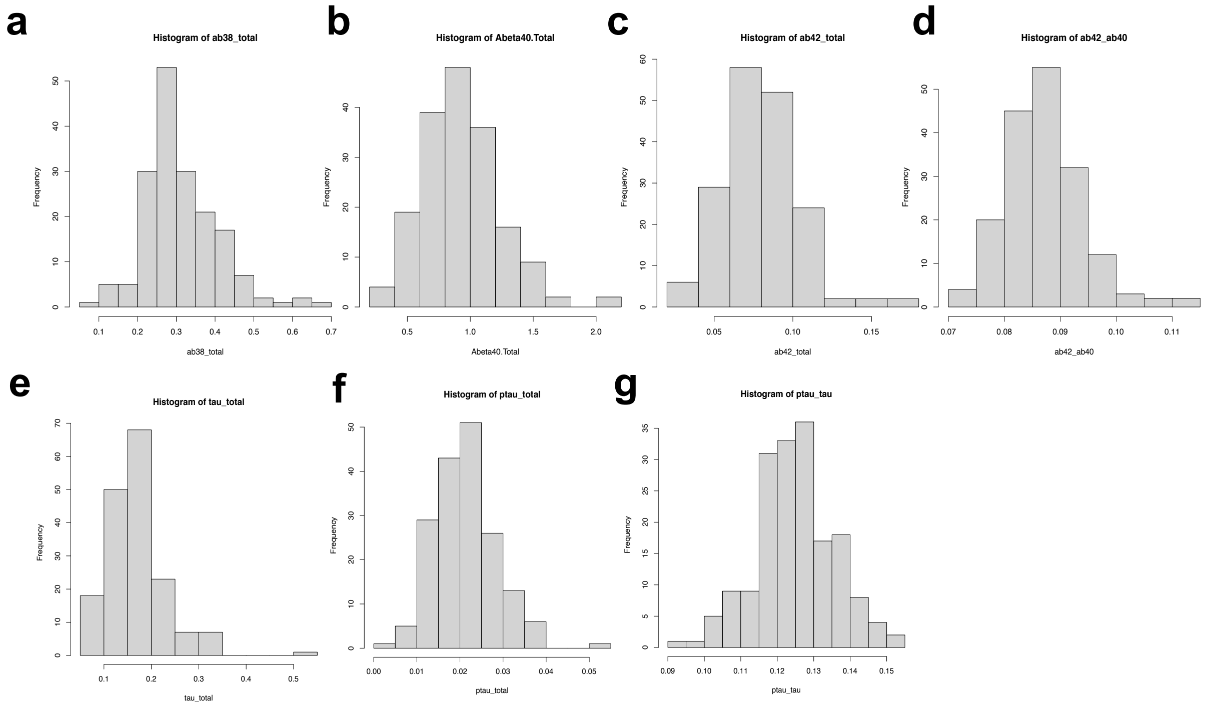


**Supplementary Figure 6. Frequency distributions of AD endophenotype measurements.** The frequency distributions of measurements for each AD endophenotype (Aβ38, Aβ40, Aβ42, Aβ42:Aβ40, p231-tau, tau, and p231-tau:tau) found by pooling the values from all 19 shRNA targets plus controls (empty vector or no virus) for each parameter. These plots demonstrate normal and normal-like distributions for each measurement.

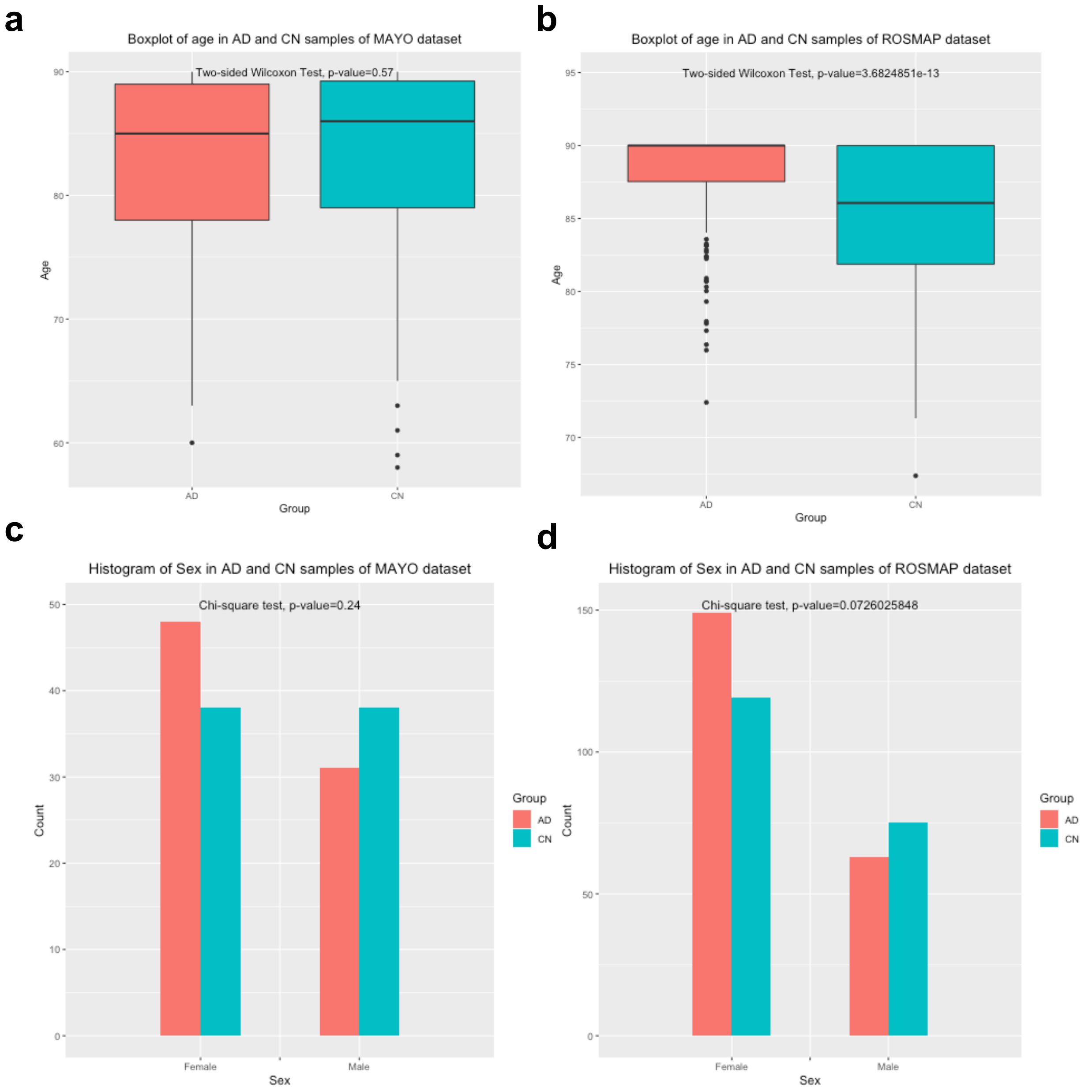


**Supplementary Figure 7. Demographical characterization in MAYO and ROSMAP.** (**a,b**) The age distribution of AD and cognitively normal (CN) samples in the MAYO (**a**) and ROSMAP (**b**) datasets, each compared using an unpaired, two-sided Wilcoxon test. There was no significant age difference in MAYO (p-value=0.57) but there was a significant difference in ROSMAP (p-value=3.68E-13). To remove this effect in ROSMAP, age was adjusted along with other covariates in the ROSMAP residuals. Box Plots in (a,b) are divided into four quartiles. The middle quartiles are represented by a box that contains 50% of the data and the median value. The upper and lower quartiles contain the maximum and minimum values and the remaining 50% of the data. (**c,d**) The sex distribution of AD and CN samples in the MAYO (**c**) and ROSMAP (**d**) datasets. A Chi-square test showed no significant difference in the sex breakdown in the MAYO (p-value=0.24) or ROSMAP (p-value=0.0726) datasets.

## Supplementary Notes

### Supplementary Note 1: MAYO diagnostic criteria

Subjects with AD each had a definite neuropathologic diagnosis according to the NINCDS-ADRDA criteria[6] and a Braak[7] neurofibrillary tangle stage of ≥4.0. Control subjects each had a Braak stage of 3.0 or less and CERAD[8] neuritic and cortical plaque densities of 0 (none) or 1 (sparse), and each lacked any of the following pathologic diagnoses: AD, Parkinson's disease, dementia with Lewy bodies, vascular dementia, progressive supranuclear palsy, motor neuron disease, corticobasal degeneration, Pick's disease, Huntington's disease, frontotemporal lobar degeneration, hippocampal sclerosis, or dementia lacking distinctive histology.

### Supplementary Note 2: ROSMAP diagnostic criteria

The ROS and MAP studies each annually administer a battery of 21 cognitive performance tests, 19 of which are in common. Alzheimer's disease status is determined by a computer algorithm based on cognitive test performance with a series of discrete clinical judgments made by both a neuropsychologist and a clinician. First, subjects are categorized as not cognitively impaired (NCI, if diagnosed without dementia), mild cognitive impairment (MCI), or Alzheimer's disease (AD). Diagnoses of dementia and AD conform to standard definitions[9]. Next, a clinician reviews all cases determined by the algorithm to render a diagnosis blinded to data collected in prior years. In addition to dementia, 5 other diagnoses are determined by this approach, including stroke, cognitive impairment due to stroke, parkinsonism, Parkinson's disease, and depression. Most of these other diagnoses are determined by self-report. Upon death, a summary diagnosis is made by a neuropsychologist blinded to post-mortem assessment. The post-mortem neuropathologic evaluation performed includes a uniform structured assessment of AD pathology, cerebral infarcts, Lewy body disease, and the other pathologies common in aging and dementia (e.g., vascular dementia or frontotemporal dementia). The evaluation procedures follow those outlined by the pathologic dataset recommended by the National Alzheimer's Disease Coordinating Center. Pathologic diagnoses of AD use NIA-Reagan and modified CERAD criteria[10], and the evaluation of neurofibrillary pathology uses Braak staging[7]. The ROS and MAP studies are both conducted by the same clinical and pathologic data collection teams, with extensive item-level harmonization allowing the data to be efficiently merged.

### Supplementary Note 3: MAYO RNAseq data collection

Temporal cortex samples from all MAYO subjects underwent RNA extraction via the TRIzol/chloroform/ethanol method, followed by DNase treatment and cleanup of RNA using Qiagen's RNase-Free DNase Set and RNeasy Mini Kit (Germantown, MD). Quantity and quality of all RNA samples were determined using the Agilent RNA 6000 Nano Kit on the Agilent 2100 Bioanalyzer system (Agilent Technologies, Santa Clara, CA). MAYO RNAseq samples were randomized across flowcells, taking into account age at death, sex, RIN, Braak stage, and diagnosis. The TruSeq RNA Sample Prep Kit (Illumina, San Diego, CA) was used for library preparation from all samples. Library concentration and size distribution were determined using an Agilent Bioanalyzer DNA 1000 Kit (Agilent Technologies). Three samples were run per flowcell lane using barcoding.

### Supplementary Note 4: MAYO genome-wide genotyping

MAYO subjects underwent whole genome genotyping using the Illumina Infinium HumanOmni2.5-8 BeadChip Kit (San Diego, CA), which delivers comprehensive coverage of

both common and rare SNP content from the 1000 Genomes Project[11] (minor allele frequency >2.5%) and provides genotypes for 2,338,671 markers.

**Supplementary Note 5: MAYO sample exclusion criteria**

Two MAYO AD subjects were excluded due to discordant sex as identified by both genotyping data and RNAseq data (deviating expression levels of Y chromosome genes with non-zero counts compared to expected expression based on recorded sex). Subjects were assessed for heterozygosity rates >3 SD from the mean. One AD sample had high heterozygosity with respect to the mean, indicating possible sample contamination, and 3 samples (2 controls and 1 AD) had low heterozygosity with respect to the mean, indicating either divergent ancestry or consanguinity; these 4 samples were also excluded from all subsequent analysis. PLINK was used to identify any sample duplicates or related pairs of subjects. Two pairs of samples were identified as >3rd degree relatives; for each pair, the sample with the lower SNP call rate was excluded. Using EIGENSOFT[12], two samples were identified as population outliers using the default parameter of >6 SD from the mean on any of the top 10 inferred axes following 5 iterations, and they were removed from further analysis. Finally, we note that we additionally excluded 2 samples during RNAseq data QC due to missing data in one or more key covariates.

**Supplementary Note 6: Evaluation of the robustness of the deconvoluted neuron-specific residual**

We evaluated the robustness of our single biomarker-derived gene expression residuals against a randomly selected subset of clean biomarkers in AD, i.e., those not affected by disease pathology and remaining cell type-specific in the AD context. We developed a 4-step procedure for the selection of this clean set of biomarkers:

1) First, we performed hierarchical clustering of all scRNAseq-derived biomarkers in the PC space (Supplementary Fig. 3b,c; Supplementary Data 12) for the MAYO and ROSMAP cohorts, respectively. This step clustered the complete list of biomarkers into small bins in AD based on their gene expression covariance (geometric distance in the PC space).
2) Next, we applied K-means clustering to group these small bins together to form larger and coherent local regions (or clusters) of biomarker genes. We used K=6 to obtain a total of 6 clusters representing the 5 cell types of interest and 1 overlapping cluster. We assigned one cell type to each cluster by calculating the percentage of biomarkers of each cell type in each cluster and assigning the cell type with the maximum percentage. Ideally, if a cluster perfectly represents one specific cell type, the percentage of that corresponding cell type is 1 and the percentages of all other cell types are 0. The cluster with a close-to-uniform distribution over the 5 major cell types (percentage around 0.2 for every cell type) was assigned as the overlapping junk cluster and was discarded in the subsequent robustness analysis because it is not cell type-specific under the AD condition. As shown in Supplementary Fig. 3d-e, with this step we identified 5 clean clusters each representing a cell type of interest for further analysis.
3) We next removed outlier biomarkers from each cluster by calculating the geometric center and standard deviation (SD) of the gene expression along each cluster's first and second PC, using 1xSD as the cutoff.
4) Finally, we removed any biomarkers overlapping with the latest Agora targets (608 genes) to remove any potential residual influence of AD pathology from each cluster. The final clusters of selected biomarkers for each cell type represent true cell type-specific biomarkers in the AD context and are shown in Supplementary Fig. 3f-g.

We note that our procedure to determine AD-independent cell type-specific biomarkers was purely data-driven, i.e., the position of our original single-biomarker genes (*ENO2, CD68, CD34,*

*GFAP,* and *OLIG2*) did not influence any step of this selection process. We also note that the selected astrocyte biomarkers still slightly overlap with endothelial and oligodendrocytic biomarkers in the MAYO and ROSMAP cohorts, respectively (Supplementary Fig. 3f-g). However, this intrinsic noise is likely attributed to confounding biological factors such as interactions between these cell types in the AD brain. This intrinsic noise is also expected and consistent with that observed in scRNAseq-derived cell type biomarkers in AD, such as the scRNAseq biomarker study[13] also using the ROSMAP cohort which showed significant overlap between astrocyte and oligodendrocyte markers (Supplementary Data 3).

Finally, using the resulting clean biomarker clusters, we randomly subsampled any number of biomarkers 1000 times and plugged them into the same deconvolution procedure as described in the Methods section "Deconvolution of RNAseq data into neuron-specific expression residuals", in order to calculate 1000 simulated residuals. We then assessed the Pearson correlation between our single-biomarker derived residuals and the 1000 simulated residuals, finding a mean correlation of 0.767 for MAYO and 0.722 for ROSMAP (Supplementary Fig. 3h-i). Based on these results, we can conclude that our single-biomarker derived residuals, including our neuron-specific residual, are robust against randomly selected cell type-specific biomarker genes that are independent of AD.

### Supplementary Note 7: Impact score

To rank the 1,563 predicted key drivers based on impact score, we calculated the following metrics:

a) **KDA_unit_score**. For each network, we performed key driver analysis (KDA). We defined different target gene lists for KDA with several gene sets: i) the overlap between DE genes and selected modules, recording the number of overlapping gene sets nominating a given gene as a key driver; ii) the selected modules, recording the number of modules nominating a given gene as key driver; and iii) the DE gene set, whose value was 1 or 0, indicating whether (1) or not (0) DE genes nominated a given gene as a key driver.
b) **KDA_score_sum**. We summed the 3 KDA_unit_score values described in a).
c) **KDA_position_sum**. We found the non-zero sum of the frequency of the 3 values in a).
d) **Normalized_priority_score**. For each network, we sorted the key drivers first according to KDA_position_sum in descending order and then according to KDA_score_sum in descending order. In each network, we then calculated the normalized_priority_score by dividing each key driver's rank by the maximum rank for the corresponding network.
e) **Replication_count**. We recorded the number of networks from which a key driver was derived.
f) **Avg_priority_score**. We calculated the averaged priority score per key driver across all networks by dividing normalized_priority_score by replication_count.
g) **Avg_DE_R**. Since a gene could appear in multiple KDA files and network files (e.g. gene A appearing in 5 KDA files and 7 networks), we selected each corresponding network from which a given key driver was derived, retrieved all downstream members in the networks of the key driver, and calculated the following metrics: i) DE_reach, the percentage of corresponding DE genes covered by the gene's downstream effectors, describing an overall overlap between downstream effectors of a key driver and DE genes; ii) the number of DE genes (N) and a modified version of count (R) for each layer of a downstream subnetwork, where the modified count (R) was computed as following: for a given DE gene, if it had X parent nodes and among those X parents, Y of them were not downstream members of the given key driver, we would say the burden factor

of the DE gene for the key driver is Y/X. Thus, for each layer in the downstream sub-network, if the layer contained Z DE genes, then N=Z and R=Z minus the sum of burden factors over all Z DE genes for the layer; iii) R.coeff, the coefficient of a linear model, where the response variable is a vector of the normalized (by number of layers) cumulative sum of R for each layer, thus describing the cumulative percentage of parents that are also DE genes between the first layer and the current layer (value between 0 and 1), and the predictor variable is the layer index. The higher this coefficient, the more impact a key driver has on the downstream DE genes; iv) impact_per_network score of a key driver, which was calculated as DE_reach*R.coeff and is a joint descriptor of the overall downstream-DE gene overlap and local DE percentage of every layer in the downstream sub-network of each key driver. Note that the above DE_reach and R.coeff values are calculated per network; and finally v) Avg_DE_R, the averaged value of impact_per_network for a key driver over all the networks to find its overall impact across all networks, since individual key drivers may be identified by multiple networks.

**h)** **Impact_score**. Finally, the impact score of a key driver was calculated as (1-avg_priority_score)*Replication_count*Avg_DE_R. We ranked the key drivers in descending order.

**Supplementary Note 8: Robustness score**

To rank the 1,563 predicted key drivers based on robustness score, we calculated the following metrics:

a) **Dataset_Count**. We calculated how many datasets, i.e., MAYO and ROSMAP cohorts, by which a key driver was replicated.
b) **Geneset_Count**. We calculated how many types of gene sets, i.e., expanded or seeding gene sets, by which a key driver was replicated.
c) **Avg_DE_R score**. This score was calculated in the same manner as described above (step g of Impact Score).
d) We ranked the key drivers according to robustness first by Dataset_Count in descending order, then ranked by Geneset_Count in descending order, and lastly ranked by Avg_DE_R score in descending order.

**Supplementary Note 9: iN media formulations**

a) **KSR media**: Knockout DMEM (Gibco), 15% KOSR (Invitrogen, Thermo Fisher Scientific), 1x MEM-NEAA (Invitrogen), 55 μM beta-mercaptoethanol (Invitrogen), 1x GlutaMAX (Life Technologies, Thermo Fisher Scientific).
b) **N2B media**: DMEM/F12 (Life Technologies), 1x GlutaMAX (Life Technologies), 1x N2 supplement B (STEMCELL Technologies), 0.3% dextrose (D-(+)-glucose, Sigma-Aldrich).
c) **NBM media**: Neurobasal Medium (Gibco), 0.5x MEM-NEAA (Invitrogen), 1x GlutaMAX (Life Technologies), 0.3% dextrose (D-(+)-glucose, Sigma-Aldrich).

## Supplementary References


1 Zhang, Y. *et al.* Purification and Characterization of Progenitor and Mature Human Astrocytes Reveals Transcriptional and Functional Differences with Mouse. *Neuron* **89**, 37-53, doi:10.1016/j.neuron.2015.11.013 (2016).

2 Lake, B. B. *et al.* Neuronal subtypes and diversity revealed by single-nucleus RNA sequencing of the human brain. *Science* **352**, 1586-1590, doi:10.1126/science.aaf1204 (2016).

3 Zeisel, A. *et al.* Brain structure. Cell types in the mouse cortex and hippocampus revealed by single-cell RNA-seq. *Science* **347**, 1138-1142, doi:10.1126/science.aaa1934 (2015).

4 Darmanis, S. *et al.* A survey of human brain transcriptome diversity at the single cell level. *Proc Natl Acad Sci U S A* **112**, 7285-7290, doi:10.1073/pnas.1507125112 (2015).

5 Zhang, Y. *et al.* An RNA-sequencing transcriptome and splicing database of glia, neurons, and vascular cells of the cerebral cortex. *J Neurosci* **34**, 11929-11947, doi:10.1523/JNEUROSCI.1860-14.2014 (2014).

6 McKhann, G. *et al.* Clinical diagnosis of Alzheimer's disease: report of the NINCDS-ADRDA Work Group under the auspices of Department of Health and Human Services Task Force on Alzheimer's Disease. *Neurology* **34**, 939-944, doi:10.1212/wnl.34.7.939 (1984).

7 Braak, H. & Braak, E. Neuropathological stageing of Alzheimer-related changes. *Acta Neuropathol* **82**, 239-259, doi:10.1007/BF00308809 (1991).

8 Mirra, S. S. *et al.* Interlaboratory comparison of neuropathology assessments in Alzheimer's disease: a study of the Consortium to Establish a Registry for Alzheimer's Disease (CERAD). *J Neuropathol Exp Neurol* **53**, 303-315, doi:10.1097/00005072-199405000-00012 (1994).

9 Bennett, D. A. *et al.* Religious Orders Study and Rush Memory and Aging Project. *J Alzheimers Dis* **64**, S161-S189, doi:10.3233/JAD-179939 (2018).

10 Mirra, S. S. *et al.* The Consortium to Establish a Registry for Alzheimer's Disease (CERAD). Part II. Standardization of the neuropathologic assessment of Alzheimer's disease. *Neurology* **41**, 479-486, doi:10.1212/wnl.41.4.479 (1991).

11 Genomes Project, C. *et al.* A global reference for human genetic variation. *Nature* **526**, 68-74, doi:10.1038/nature15393 (2015).

12 Patterson, N., Price, A. L. & Reich, D. Population structure and eigenanalysis. *PLoS Genet* **2**, e190, doi:10.1371/journal.pgen.0020190 (2006).

13 Mathys, H. *et al.* Single-cell transcriptomic analysis of Alzheimer's disease. *Nature* **570**, 332-337, doi:10.1038/s41586-019-1195-2 (2019).
