# Supplementary material for: Predictive network analysis identifies JMJD6 and other potential key drivers in Alzheimer’s disease
Source: Commun Biol. 2023 May 15;6:503. doi: 10.1038/s42003-023-04791-5 (PMC10185548; doi:10.1038/s42003-023-04791-5)
Supplement: Supplementary file 2 — Description of Additional Supplementary Files [file 42003_2023_4791_MOESM2_ESM.docx]

1 Description of Additional Supplementary Files

2

1. **File name:** Supplementary Data 1
2. **Description:** Neuron-specific DE signatures associated with AD in the MAYO and ROSMAP RNAseq datasets. As noted in the main text, there are 2,097 significant DE genes overlapping between the two datasets (Fisher’s exact test, odds ratio=3.9784, p-value<2.2E-16).
3. **File name:** Supplementary Data 2
4. **Description:** Significantly enriched pathways associated with neuron-specific DE gene signatures in the MAYO and ROSMAP RNAseq datasets, indicating dysregulated biological processes in AD. Pathway enrichment was assessed using Human ConsensusPathDB.
5. **File name:** Supplementary Data 3
6. **Description:** Robustness analysis of our neuron-specific DE signatures in the MAYO and ROSMAP cohorts against scRNAseq-derived DE signatures in ROSMAP. Here, pair-wise enrichment analysis was performed among the scRNAseq-derived DE gene signatures in the ROSMAP dataset for excitatory neurons (EX), inhibitory neurons (IN), astrocytes (AST), oligodendrocytes (OLI), oligodendrocyte progenitor cells (OPC), and microglial cells (MIC), using Fisher’s exact test with FDR<0.05.
7. **File name:** Supplementary Data 4
8. **Description:** Robustness analysis of our neuron-specific DE signatures in the MAYO and ROSMAP cohorts against scRNAseq-derived DE signatures in ROSMAP. Here, pair-wise enrichment analysis was performed to compare each of our neuron-specific DE signatures (MAYO and ROSMAP) against the scRNAseq-derived signatures for excitatory neurons (EX), inhibitory neurons (IN), astrocytes (AST), oligodendrocytes (OLI), oligodendrocyte progenitor cells (OPC), and microglia (MIC) in the ROSMAP dataset, using Fisher’s exact test with FDR<0.05.
9. **File name:** Supplementary Data 5
10. **Description:** List of cis-eQTL genes in the MAYO and ROSMAP datasets.
11. **File name:** Supplementary Data 6
12. **Description:** Significantly enriched biological pathways associated with gene modules in the MAYO and ROSMAP neuron-specific co-expression networks.
13. **File name:** Supplementary Data 7
14. **Description:** Statistical analyses and summary of Aβ and tau data from human iNs following shRNA knockdown of each of the 19 prioritized key driver targets.
15. **File name:** Supplementary Data 8
16. **Description:** Summary of the overlap of the 10 validated knockdown targets with DE and *cis*-eQTL genes in the MAYO dataset.
17. **File name:** Supplementary Data 9
18. **Description:** Summary of the overlap of the 10 validated knockdown targets with DE and cis-eQTL genes in the ROSMAP dataset.
19. **File name:** Supplementary Data 10
20. **Description:** DE analysis of gene expression from RNAseq data from human iNs following shRNA knockdown of each of the 10 AD endophenotype-modulating key driver targets.
21. **File name:** Supplementary Data 11
22. **Description:** Significantly perturbed pathways associated with DE signatures of each of the 10 AD endophenotype-modulating targets in human iNs.
23. **File name:** Supplementary Data 12
24. **Description:** Merged cell type-specific biomarker lists for neurons, microglia, astrocytes, endothelial cells, and oligodendrocytes derived from existing scRNAseq studies (referenced in 1-5) from control human brains.
25. **File name:** Supplementary Data 13
26. **Description:** Source data underlying Fig. 2c.
27. **File name:** Supplementary Data 14
28. **Description:** Source data underlying Fig. 3b.
29. **File name:** Supplementary Data 15
30. **Description:** Source data underlying Fig. 4c-d.
31. **File name:** Supplementary Data 16
32. **Description:** Source data underlying Fig. 5a-g. 35

36
